# HIV Treatment as Prevention: Modelling the Cost of Antiretroviral Treatment—State of the Art and Future Directions

**DOI:** 10.1371/journal.pmed.1001247

**Published:** 2012-07-10

**Authors:** Gesine Meyer-Rath, Mead Over

**Affiliations:** 1 Center for Global Health and Development, Boston University, Boston, Massachusetts, United States of America; 2 Health Economics and Epidemiology Research Office, Department of Medicine, Faculty of Health Sciences, University of the Witwatersrand, Johannesburg, South Africa; 3 Center for Global Development, Washington, District of Columbia, United States of America; Massachusetts General Hospital, Harvard Medical School, United States of America

## Abstract

Policy discussions about the feasibility of massively scaling up antiretroviral therapy (ART) to reduce HIV transmission and incidence hinge on accurately projecting the cost of such scale-up in comparison to the benefits from reduced HIV incidence and mortality. We review the available literature on modelled estimates of the cost of providing ART to different populations around the world, and suggest alternative methods of characterising cost when modelling several decades into the future. In past economic analyses of ART provision, costs were often assumed to vary by disease stage and treatment regimen, but for treatment as prevention, in particular, most analyses assume a uniform cost per patient. This approach disregards variables that can affect unit cost, such as differences in factor prices (i.e., the prices of supplies and services) and the scale and scope of operations (i.e., the sizes and types of facilities providing ART). We discuss several of these variables, and then present a worked example of a flexible cost function used to determine the effect of scale on the cost of a proposed scale-up of treatment as prevention in South Africa. Adjusting previously estimated costs of universal testing and treatment in South Africa for diseconomies of small scale, i.e., more patients being treated in smaller facilities, adds 42% to the expected future cost of the intervention.

## Introduction

Informed by biological plausibility [1], observational studies [2], and a trial [3] showing that ART reduces transmission of HIV within heterosexual serodiscordant couples, recent modelling papers [4]–[6] have projected the reduction in HIV incidence and the impact on health care costs that would follow from achieving close-to-universal coverage with HIV testing and ART. These papers argue that sufficiently universal ART coverage would eventually pay for itself by suppressing HIV incidence and therefore averting the future need for HIV care, including ART. Other papers in the July 2012 *PLoS Medicine* Collection, “Investigating the Impact of Treatment on New HIV Infections” analyse the sensitivity of the projected population-level incidence reductions to the structure and assumptions of an epidemiological projection model [7]–[9]. This paper focuses on the cost side of such projection models. We begin with a general discussion of cost accounting identities versus flexible cost functions. Then we review the available literature on modelled estimates of the projected cost of ART provision, including ART for prevention, with a focus on identifying determinants authors have included, implicitly or explicitly, in their assumed cost function for ART service delivery. We then discuss the evidence for a number of such cost determinants. Finally, we present an example of a flexible cost function used to explore how economies of scale might affect the costs of scaling up ART in South Africa. A second paper focussing on economic evaluation in this collection further discusses how operational and effectiveness issues in scaling up ART for prevention will affect its cost-effectiveness [10].

## Cost Accounting Identities versus Flexible Cost Functions

Just as most epidemiological projection models include a functional representation of epidemiological concepts such as the force of infection, cost projection models include a function or a set of functions to characterise the relationship between the total cost of ART service delivery and various determinants of cost, such as the number of patients on treatment, the stage in their disease at which they were recruited, and the ART regimen they receive. Most existing cost projections assume a single constant unit cost per patient-year, or per patient-year on a certain regimen, across large populations and often extended projection periods. A somewhat more complex approach is to assume a single unit cost for each of a set of services received by an HIV-positive patient, such as a unit cost for each type of laboratory test or outpatient visit or inpatient day, and then multiply these unit costs by an estimate of the number of each of these services per patient-year and by the number of patient-years delivered in a year. We call such an equation an accounting identity and designate a total annual cost so defined as an accounting identity cost function, TC_AI_. In its simplest form such a cost function can be written as
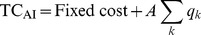
(1)where *k* indexes the facilities delivering ART, *q_k_* represents the output of facility *k* in a single time period, typically a year, and *A* is the average variable cost per patient-year. Cost accounting identities impose the discipline of arithmetical consistency on discussions of budgets, costs, expenditures, and efficiency, and predict future expenditures over the short run. They are a natural approach when estimating how much delivery of a service “should” cost. (See the discussion of the distinction between “normative” and “positive” cost functions in Text S2.) As such, they are often sufficient for capturing the impact of incremental policies, e.g., an extension of a health care intervention to a slightly larger proportion of the same population by increasing coverage.

However, cost accounting identities cannot be used to predict how costs will change when civil servants, managers, providers, and patients have an opportunity to adjust service delivery by, for example, substituting one input for another, or changing the scale and scope of operations, eligibility criteria, task shifting, or the deployment of supply- or demand-side incentives. We argue that, as a result, cost accounting identities are too rigid to model large-scale changes over periods of more than a few years—such as those required to achieve the HIV prevention benefits of ART. For these purposes, a more flexible cost function such as
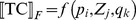
(2)can provide a more plausible characterisation and projection of total annual costs. In Equation 2, *p* and *Z* are vectors representing, respectively, the set of relevant input prices and all other policy and environmental determinants of cost, many of which we discuss in this paper. The notation *f*(…) stands in for a flexible functional form chosen either to fit the data or, when data is lacking, to fit the analysts' assumptions (see Text S2 for more details). For simplicity, in both Equation 1 and 2 we have suppressed the time subscripts, but in a more formal development, time might itself influence price, output, or other policy determinants.

## The Use of Cost Functions in Published Modelled Economic Analyses of ART

In order to determine the current state of the art, we reviewed the available literature on modelled estimates of the projected cost of ART provision to a variety of eligible populations, including ART for prevention. We searched eight databases (PubMed, HealthSTAR, POPLINE, EconLit, HEED, Web of Knowledge [Science and Social Sciences], Embase and CAB Health) for the years 1988–2011 using any combination of the terms cost*, econ*, and *HIV* or *AIDS*. We supplemented the identified articles by reviewing the reference lists of identified articles, additional review articles, and grey literature (slides, conference proceedings, books, and manuals). We included all articles in any language that contained modelled cost data of any kind as well as ART as an intervention, except where it was used for the prevention of mother-to-child transmission only. Abstracts and articles in all languages (English, Italian, Spanish, French, and German) were read in full by the first author, who made the decision whether to include the article in the review. We excluded editorials and letters, articles without quantitative data, and articles that did not include a modelled estimate, such as papers reporting cost data from a single site. The last have been reviewed repeatedly in the past [11]–[15]. We reviewed the included articles with regards to their economic evaluation method, the type of model used, their time horizon, the outcome metric and result, and whether the input cost (often in the form of average per patient cost per unit time) was constant or had been varied by determinants such as types of regimens used, health state, time on treatment, and mode of delivery, in either the main or the sensitivity analysis.

We identified 45 published articles, one conference abstract, and four reports on modelled economic analyses of ART provision worldwide (Table 1; Text S1). Thirty-eight analyses were for single countries, four were for wider regions, and eight were global. Five analyses, all for single countries, specifically considered the impact of ART on HIV transmission; we discuss these separately.

**Table 1:** Overview of the methods and results of previously published modelled economic analyses of antiretroviral treatment.

Category	Number of papers	Number with No Variation in Input Cost in Main Analysis	Number with Unit Cost Held Constant within Each Determinant				Number with Sensitivity Analysis			Results in 2011 US Dollars		
			Regimen	Health State	Time on Treatment	Other Variables	Done (of Which Probabilistic)	Includes Drug Cost	Includes Other Cost	Cost per Life Year Saved	Cost per QALY Gained	Total Annual Cost
**No impact of treatment on transmission assumed**												
Single country, high income	24	2	18	23	1	US state: 1; medication payer: 1	20–21^a^ (1)	14	13	9,027–84,882^b^; 13,781–42,944^c^; cost savings: 71,111^d^	20,885–40,279^c^; 16,430–295,113^d^	
Single country, low/middle income	9	1	5	6	2	Inpatient cost by level of care: 1; mode of delivery (public versus private): 1	5 (1) plus 1 scenario analysis	6	4	40–2,540^d^	1,098–18,851^d^	
Regional	4	1–2^e^	2	0	0	0	1 (0)	1	1			2.3 billion^d^–9.3 billion^d^
Global	8	3	2	1	0	Access to CHAI prices: 1; GNP per capita: 1; shift to primary care and cheaper diagnostics: 1	4 (1)	2	3			1.8 billion^d^^,^ f–176 billion^d^^,^ g
**Impact of treatment on transmission assumed**												
Single country, high income	1	0	0	1	0	0	1 (0)	0	0		20,542–22,649^d^	
Single country, low/middle income	5	0	3	3	1	Unstructured versus structured treatment provision: 1	4 (0)	2	2		Cost savings: 4,594^d^	

a One publication ([28]; in abstract format) has no information on whether sensitivity analysis was conducted.

b For zidovudine monotherapy.

c For dual therapy.

d For highly active ART.

e One study [50] does not supply enough information on ART input cost to know whether it is constant.

f Analysis from 2011, based on country-level cost data.

g Analysis from 1997, based on high-income country cost data extrapolated worldwide.

CHAI, Clinton HIV/AIDS Initiative; GNP, gross national product; QALY, quality-adjusted life year.

Thirty-three analyses modelled ART programmes within a single country, without considering the transmission impact of ART [16]–[48]. Most of the 24 high-income-country analyses compared the incremental cost and effectiveness of a new drug regimen with that of an older one [22],[24]–[26],[33],[36]–[39]. Amongst the nine low- and middle-income-country (LMIC) analyses, six analyses focussed on the choice of eligibility criteria [40]–[45],[47],[48]. One analysis compared ART with no ART [43], one, first-line treatment with first- and second-line treatment [40], and one, different regimens for women previously exposed to single-dose nevirapine as part of prevention of mother-to-child transmission [47].

In terms of the use of cost functions, most of these single-country papers varied input cost (i.e., the cost per patient per unit of time) by protocol-related variables such as treatment regimen, health state (defined by the absence or presence of symptoms, opportunistic infections, AIDS-defining diseases, and/or CD4 cell count levels), and/or time on treatment (see Table 1). Only two papers, both of them on LMICs, varied cost by level of care (secondary versus tertiary) [43] or mode of health care provision (public versus private) [44]; none of the papers varied per patient cost by scale or other programmatic variables.

The four regional studies [49]–[52] all focussed on sub-Saharan Africa (with one study [52] additionally including Southeast Asia). These studies modelled the cost of defined increases in ART coverage from a low baseline [49],[50] and the cost effectiveness of ART provision through the specific setting of an antenatal care clinic [51]. One paper used the same constant input cost for all patients [52]; two papers varied input cost by regimen [49],[52]. None of the papers varied per patient cost by any other variables.

The eight global studies, published between 1997 and 2011, describe a clear evolution in both data availability and modelling technique [53]–[60]. The older analyses estimate cost based only on the number of HIV-positive people from a number of sources, varying assumptions of ART coverage at baseline, with costs based on guidelines and prices from high-income countries [53],[54]. Later analyses model global cost under concrete programmes, such as the World Health Organization's 3 by 5 initiative [57] and the Global Fund to Fight AIDS, Tuberculosis and Malaria [56],[58], based on per patient cost estimates from relevant LMICs and more advanced epidemiological models of the number of patients in need of ART, such as the Spectrum model [58],[59] and the Resource Needs Model [60]. Three of the eight global analyses used constant input costs for all patients [53]–[55]; two varied input cost by regimen [57],[58], and one additionally by health state [58]. One study included the impact of access to pool procurement prices negotiated by the Clinton HIV/AIDS Initiative on per patient cost [57], one varied drug prices by per capita gross national product [56], and one assumed a reduction of per patient cost of 65% by 2020 as a result of task shifting and cheaper point-of-care diagnostics [60]. No other cost determinants were considered.

Five studies between 2006 and 2011 that analysed the cost of ART for a single country included an impact of treatment on HIV transmission and, hence, on the number of future infections and future cost [4],[5],[61]–[63]. Three of these analyses were cost-effectiveness analyses of different strategies of eligibility and coverage [61]–[63]; two were analyses of the cost impact and cost benefit of earlier treatment initiation, including universal testing and treatment [4],[5]. With respect to cost functions, three of the analyses varied input cost by regimen [4],[5],[63], three by health state [61]–[63], and one by time on treatment [62]; additionally, one analysis varied input cost by whether treatment was administered in a structured way in the public sector or an unstructured way in the private sector [62]. No other variation in cost was considered.

## Potential Determinants of a Flexible Cost Function

As summarised above, most modelled estimates of the projected cost of ART provision to date have used cost accounting identities, with minimal use of cost functions. If a more flexible cost function is chosen for modelling the future cost of ART over several decades, which variables should be included in this function? Here and in Table 2, we review the evidence for some possible determinants of the cost of ART provision.

**Table 2:** Schematic summary of determinants of the cost of ART provision.

Determinant	Metric	Direction and Size of Change in Cost	Direction of Change with Scale	Open to Direct Manipulation?
Treatment characteristics: regimens, health states, time on treatment	Median CD4 cell count under ART; distribution into first line/second line; proportion of cohort with CD4 <50 cells/µl	↓↓	↑	No
Factor prices	Cost per input	↓/↑	↓	(Yes)
Scale	Number of patients; number of ART clinics	↓↓, then ↑	—	(Yes)
Experience of facility and programme	Total patient-years of treatment	↓	↑	No
Scope (facility type) and distribution into care sectors	Proportion treated in primary- versus secondary- versus tertiary-level clinics versus stand-alone clinics; proportion treated by public versus private (for-profit and not-for-profit)	↓/↑	↑	Yes
Quality of care	Retention ± clinical improvement (weight, CD4 cell count, viral load)	↑, then ↓	↓	Yes
Technical efficiency: incentives, supervision, and technical change	Provider payments as a function of output or outcome; frequency/intensity of supervision/training; doctor/nurse ratio or protocol selection	↓, except technical change:?	?	Yes

### Treatment Characteristics: Regimens, Health States, Time on Treatment

Most reviewed papers recognised that more complex cases of any disease engender higher treatment costs. Modellers addressed this by assuming a unit cost that varied by treatment regimen, health state, or time on treatment. These are important cost determinants, since the cost of a national programme will be largely defined by the distribution of the national treatment cohort into first- and second-line regimens (with second-line regimens being much more expensive in most countries) [64] and into CD4 cell count strata associated with different disease burden and cost. Likewise, an analysis of hospitalisation frequency and cost in the same patients before and after ART initiation found the cost of hospitalisation per patient-year in patients with CD4 cell count <100 cells/µl to be ten times higher than in patients with CD4 cell count >350 cells/µl [65] (see also [66]–[68]). However, we argue that these characteristics are not the only ones that input cost should vary by, and their relevance for total cost might be overwhelmed in situations of rapid scale-up or large-scale changes to programme delivery such as task shifting to lower levels of facilities and health care cadres.

### Factor Prices

The prices of factors of production, including labour, supplies, utilities, transportation, equipment, and buildings, clearly affect the cost of health services. By varying input cost by treatment regimen and, in some cases, also changing the cost of laboratory tests over time, most of the reviewed analyses have taken factor prices into account. And for good reason: the cost of antiretroviral drugs—in many countries the largest component of the cost of ART provision—has changed dramatically over the last ten years, especially for LMICs. By October 2000, the prices of antiretroviral drugs in resource-constrained settings had fallen by 90% on average [69], owing largely to the increased availability of generically manufactured drugs from three Indian companies and the possibility of importing these drugs in parallel with patent-protected drugs under the World Trade Organization Agreement on Trade-Related Aspects of Intellectual Property Rights [70]. The price of the non-generic version of the most common first-line drug combination (stavudine+lamivudine+nevirapine) dropped by 93% from US$10,439 to US$727 between June 2000 and September 2001 [71]. Even though the price of the regimen fell by another 54% between 2001 and 2008, the scope for further reductions in the price of antiretrovirals is assumed to be limited, shifting the focus to the cost of other factor prices such as service delivery, laboratory tests, and overheads.Reductions in all of these are targeted by UNAIDS's Treatment 2.0 initiative [72].

### Scale

As mentioned, none of the reviewed papers considered an impact of scale, i.e., the size or coverage of the programme, on cost, despite the dramatic increases in scale modelled by some of the papers—especially those analysing the cost of treatment for prevention [4],[5]. This stands in contrast to much of economic theory, which assumes a U-shaped relationship between scale and average cost, with cost per unit of output at first decreasing as quantities of output increase, because inputs (e.g., staff) are shared to produce an increasing number of outputs (e.g., patients seen). When scaling up further, beyond a certain number of outputs, new inputs will be required, leading to increasing average cost for large facilities or broadly expanded programmes. Scale economies seem plausible in ART service delivery because the cost of some functions of an ART treatment site, such as building maintenance, personnel management, and the transportation of supplies, will increase in more direct proportion to the number of sites than to the number of patients each one serves. This means that at the site level, increasing the number of patients generates a less than proportionate increase in cost.

Only a few programmes have produced data that have allowed this relationship to be examined empirically. Economies of scale have been found in HIV prevention programmes [73]–[75] and in the modelled cost of hygiene outreach interventions, the latter showing a U-shaped relationship between coverage and average or marginal cost [76]. The worked example below and Text S2 provide more discussion of the concept and application of scale economies.

### Experience of Facility and Programme

The implementation of most interventions is traditionally assumed to benefit from “learning by doing”, which results in reductions in average cost. Since this learning often coincides with scale-up, this relationship is not always easy to distinguish from the reduction of average cost with scale mentioned above. In an analysis of data from ART clinics supported by the US President's Emergency Plan for AIDS Relief, Menzies et al. found that median per patient cost across a number of sites in different countries decreased with each successive six-month period from the start of the ART programme at each site [77], with the biggest decrease between the first and the second six-month periods. The potential effect on cost of increased facility and programme experience over time was not considered in any of the reviewed papers.

### Scope (Facility Type) and Distribution into Care Sectors (Private versus Public)

**Figure 1:**
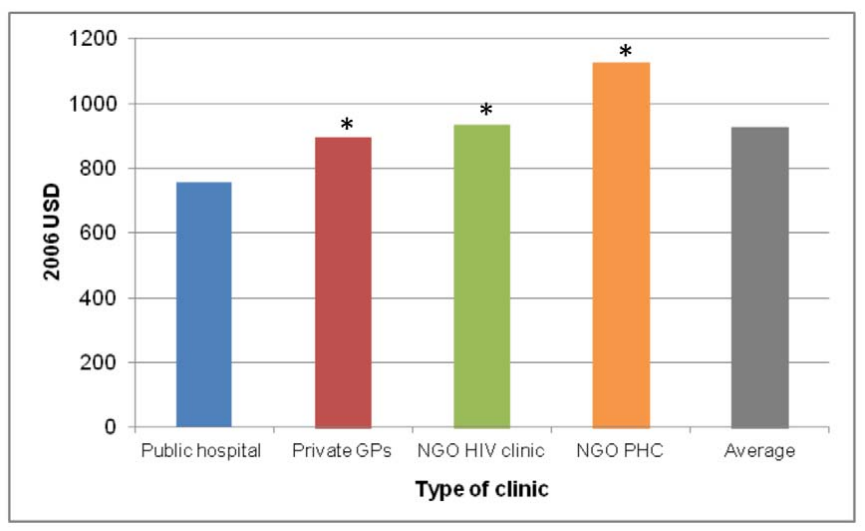
Annual per patient cost of ART provision in four different settings in South Africa. Based on [78]. *, difference from public hospital significant at *p*<0.05. GPs, general practitioners; PHC, primary health care clinic; USD, US dollars.

As with scale, the cost of a national ART programme will also be affected by a change in the scope of ART provision, i.e., the type of facilities (e.g., primary health care clinics versus specialised ART clinics at secondary- or tertiary-level hospitals) and whether or not they are in the public or the private sector, with the private sector further divided into for-profit and not-for-profit (e.g., non-governmental organisations [NGOs]). Generally, larger health care facilities, such as hospitals, can achieve economies of scope by spreading the cost of infrastructure over the production of multiple health services. Rosen et al. compared the cost of ART provision per patient-year for the first 12 months of treatment across a clinic in a public hospital, a group of private general practitioners, a private NGO-run HIV clinic, and a private NGO-run primary health care clinic in South Africa [78]. They found costs to vary significantly between sites as a result of differences in service delivery (see Figure 1). Since patient mix was comparable across three of the four sites, only a small portion of the difference in cost could be ascribed to differences in disease severity. Amongst the reviewed papers, only three included level of care as a variable determining input cost (in South Africa [43], India [44], and Thailand [63]). Future cost projections should include information on the variation of cost by level of care and mode of delivery, as well as the expected distribution of the treatment cohort between different levels and modes, especially where these are likely to change as a result of planned dramatic increases in the size of the programme.

### Quality of Care

**Figure 2:**
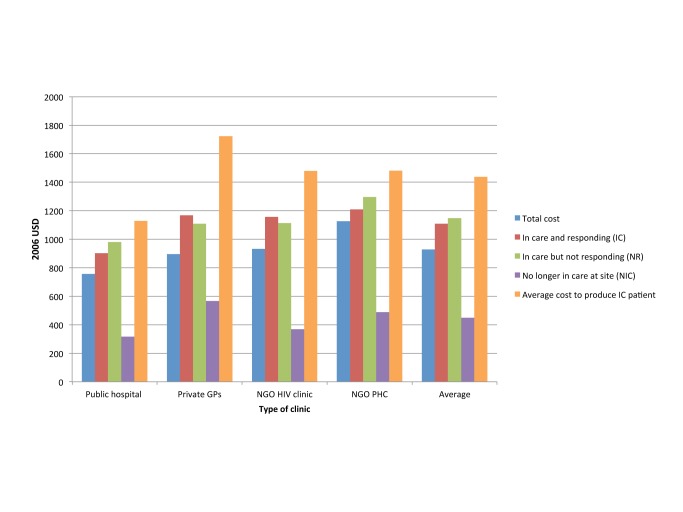
Annual per patient cost of ART provision per type of outcome in four different settings in South Africa. Based on [78]. GPs, general practitioners; PHC, primary health care clinic; USD, US dollars.

Quality of health care is notoriously difficult to measure, but in ART service delivery, a facility's success at retaining patients in treatment, and improving the patient cohort's health on average, is a reasonable proxy. The same analysis by Rosen et al. compared the cost per quality-adjusted output between the four settings, using routinely collected data (such as patient status, CD4 cell counts, viral loads, and the absence or presence of new World Health Organization stage 3 or 4 conditions) to calculate patient retention in care and response to treatment [78]. While the cost of patients who were no longer in care (i.e., had died or been lost to follow-up during the first 12 months after treatment initiation) was comparable across settings, the cost per patient in care and responding to treatment, and the cost per patient in care and not responding to treatment, was significantly different between the four clinics (Figure 2). Depending on the quality of care in each clinic, and the resulting levels of loss to follow-up and treatment failure, the additional cost per patient in care and responding was 22% and 48% of the average annual cost per patient at two sites because of resources spent on patients either leaving care or not responding to care.

### Technical Efficiency: Incentives, Supervision, and Technical Change

Technical efficiency is defined as the production of a good or service without waste, and is thus another important determinant of cost. Both public and private sector providers face constraints in the availability and quality of staff, which will affect the cost of rolling out an intervention differently at a different scale. Staffing in the public sector faces constraints such as lower wages, low work morale, and staff absenteeism, which result in low quality of care. Staffing in the private sector may not be subject to those issues to the same extent because of fee-for-service financing mechanisms, but fee-for-service mechanisms have the undesirable effect of deterring patients, especially uninsured patients, from seeking treatment [79]. Leonard and colleagues have shown that non-financial incentives such as encouragement and supervision by a peer can improve the quality of care provided by health care workers [80],[81]. As donor programmes such as the US President's Emergency Plan for AIDS Relief and its contractors relinquish direct control of patient treatment in favour of subsidies to NGOs or technical support for local government provision, the issue of management will become increasingly important as a determinant of technical efficiency and therefore costs.

Our view that programme characteristics such as scale/coverage, scope, managerial incentives, and quality/effectiveness can have important effects on the costs of ART delivery is endorsed by a second paper in this *PLoS Medicine* collection [10], which also points to the difficulty of projecting the future costs of technologies that are not yet widely used or have not even been invented. The solution to the former problem is to collect cost data on a wide range of current practices, and project future costs under the hypothesis that the technology mix will shift, e.g., towards smaller scale treatment programmes, as in the example in the next section. Projecting the costs of unknown future innovations is a less tractable problem, but arguably could best be approached by using simple flexible functions of a few fundamental variables like input prices, and allowing technical efficiency to improve according to a time trend.

## A Worked Example of a Flexible Cost Function: The Impact of Scale on the Cost of Universal Testing and Treatment

**Figure 3:**
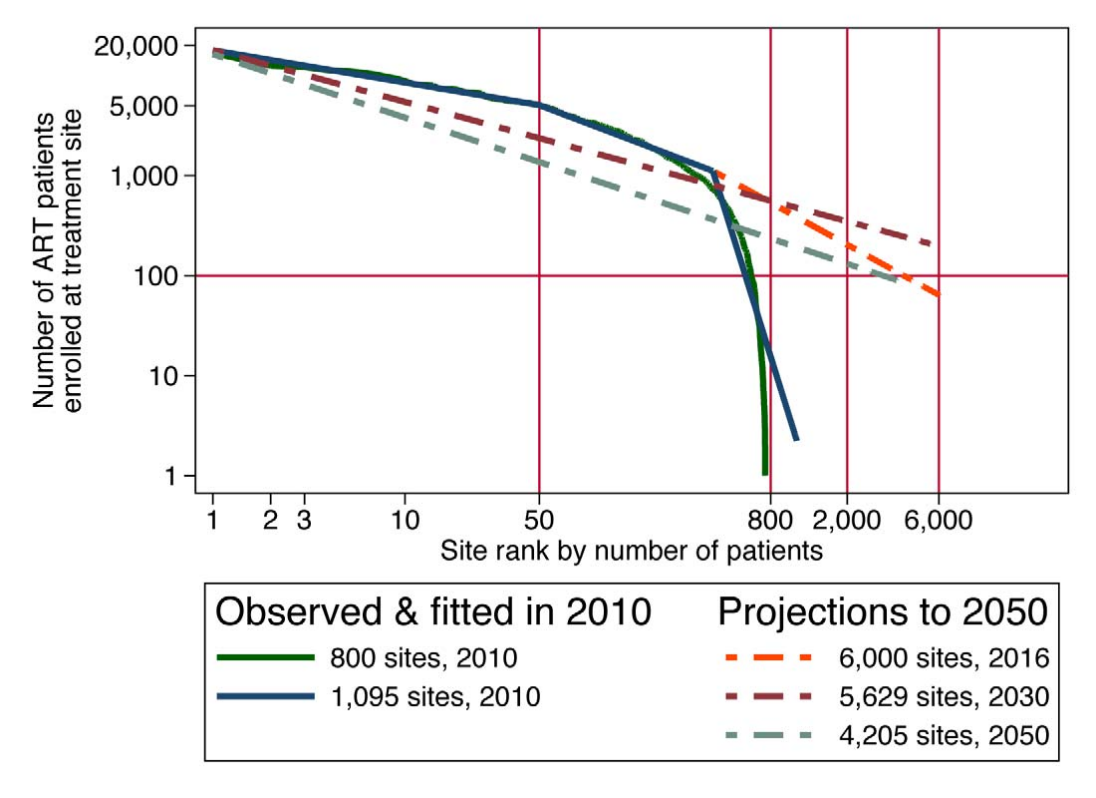
Size-rank distribution of ART facilities in 2010 and projected to future years in order to implement a universal test-and-treat strategy in South Africa.

For achieving the target coverage for universal testing and treatment in South Africa, Granich et al. [4] proposed a scale-up from 1.5 million patients on ART in June 2011 [82] to 4.1 million patients by mid-2016. While a flexible cost model of this scale-up proposal could incorporate any of the cost determinants described above, we have data on only one of these: the current size distribution of treatment facilities, i.e., scale. Since economies of scale seem likely to be a persistent feature of ART service delivery, we use this cost determinant in this example, with the hope that more of the data needed to model other potentially important cost determinants will become available in the future. We reviewed the actual size distribution of accredited ART treatment sites in South Africa in June 2010, using government and other sources (Figure 3). When the logarithm of size is charted against the logarithm of size rank, many size distributions in nature are approximately linear, following Zipf's law [83]. We hypothesize that the marked nonlinearity of the size distribution of South Africa's ART sites in 2010 was due to the recent scale-up occurring in larger sites and was temporary. If that is true, and if the largest 50 sites are assumed to retain their current patient loads during programme expansion, then expansion from 1 million patients at the beginning of 2010 to 4.1 million in 2016 would require that more sites be opened and that the scale of smaller sites be increased sufficiently to accommodate the additional patients. As a result, the size distribution of ART sites would straighten out over time. Then, as patient load subsequently contracts over time due to the hypothesized prevention success of the universal test-and-treat policy, we expect the size distribution to mature into a power law that is linear in logarithms, which first steepens, as smaller sites contract first and, once the number of enrolled patients contracts to below 1 million, contracts proportionally at all sites (see Text S2 for details).

**Figure 4:**
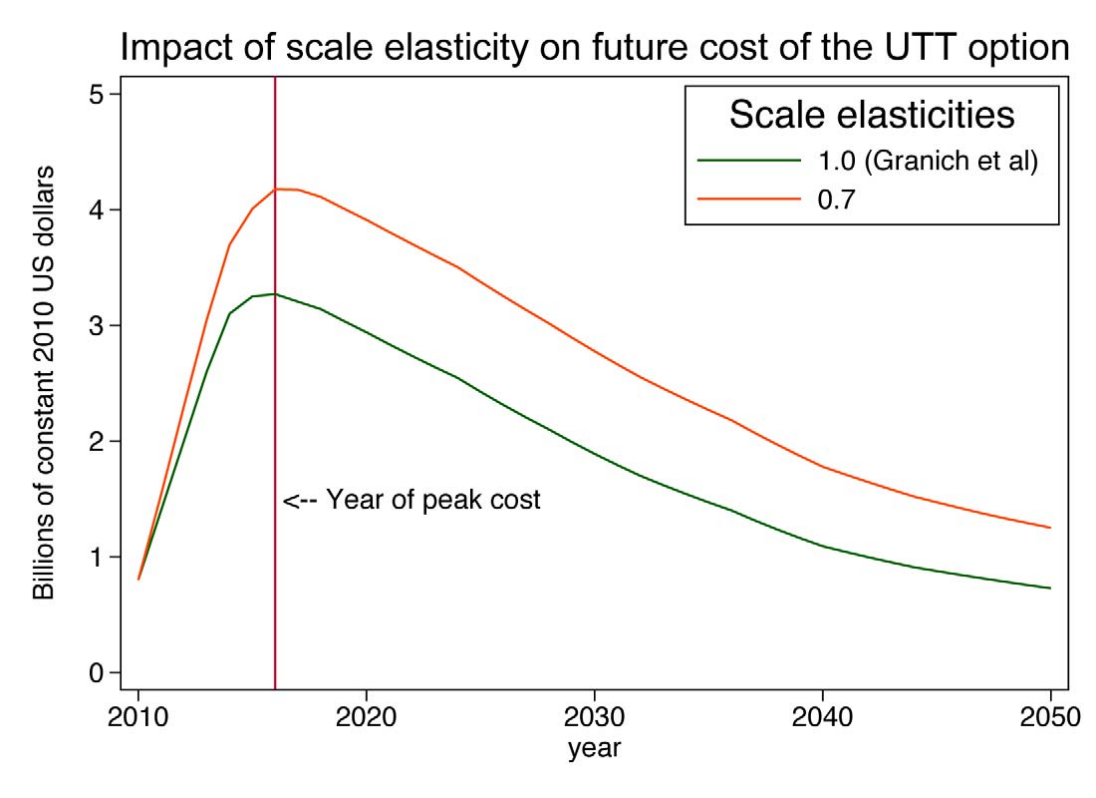
Impact of scale elasticity on future cost of a universal test-and-treat strategy in South Africa. UTT, universal testing and treatment.

Assuming a plausible size distribution of the patient load at ART sites allows us to estimate the effect that a cost function incorporating scale economies would have on the projection of total cost. Suppose that the production technology of ART services exhibits a scale elasticity of 0.7, meaning that every 10% increase in scale is associated with only a 7% increase in total cost, because of scale efficiencies. Assuming for simplicity that all economies of scale occur at the facility level, total cost (tc) for the country would be the sum of
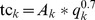
(3)over all the sites in the country, where *A_k_ = f*(*p_k_*, *Z_k_*), held constant at 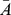 in the present analysis (US$7,600, calibrated from the known size distribution of patients and total cost per patient in 2010; Text S2 gives results for other elasticities of scale between 1.0 and 0.5). Since average cost at a site is defined as total cost at that site divided by quantity of patients at that site, the facility-specific average cost function (atc*_k_*) consistent with Equation 3 is
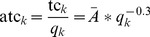
(4)Applying this cost function to the current and projected facility size distribution yields estimates of the total national cost of ART in each year of the simulation, which we compare to the Granich et al. estimates for the same scale-up scenario (Figure 4). Our assumption that the number of clinics must expand substantially to serve the estimated 4.1 million patients means that an increasing proportion of patients will be served in smaller clinics, which suffer from diseconomies of operating at small scale. In comparison to Granich's estimate of a peak annual cost of US$3.5 billion in 2016, the scale-adjusted estimate is US$4.4 billion, or 26% higher. As the number of patients moderates over time (due to Granich et al.'s assumptions of a strong population-level reduction in HIV transmission and of a concomitant 40% reduction in risky behaviour), the excess of scale-adjusted costs over accounting identity costs declines to below 20% and then rises again to 50% by the year 2050. Total accumulated cost over the 40-year period of the projection rises from US$75 billion to US$106 billion, an increase of 42%.

This example shows that the simple adjustment of the cost per patient-year for scale and a plausible pattern of distribution of patients into clinics can have a major impact on projected costs over future decades and can highlight the challenge of scaling up a treatment programme to full coverage of people outside urban areas.

## Conclusions and Recommendations for Improved Cost Projections

For modellers' projections of alternative ART scale-up scenarios to attract serious policy attention, the assumptions and structure of the cost side of these models, like those on the epidemiological side, should be plausible, supported by observational studies, and, where available, based on results from trials of the costs of alternative service delivery methods. The envisaged cost-effectiveness analyses alongside the planned large-scale trials of treatment as prevention that will be rolled out over the next years provide a historic opportunity to collect such data and allow more precise projections of the future cost of ART programmes using flexible cost functions. Text S2 provides a summary of the differences in data and algebra needed for an accounting identity versus a flexible cost function for estimating cost for an individual facility's or a country's national ART programme. Data collection on large samples of facilities should go beyond measuring the quantity and quality of ART services, to capturing the actual cost of services delivered in a sample of facilities at different levels of care and details about all of the above-listed determinants of cost. With such data on a sample of ART facilities within its own borders, a country's government and any donors supporting its HIV care programme can not only improve their projections of the long-term implications of any given commitment to antiretroviral treatment, but also model the benefits of policies to improve the cost-effectiveness of their efforts.

**Key Points**

- In modelling the projected costs of a health programme, flexible cost functions, in which costs vary with certain known or assumed determinants, provide a more plausible characterisation and projection of total annual costs than simple accounting identities.
- A review of previous models estimating the cost of ART provision indicates that while most models accounted for how costs vary with patient health status and treatment regimen, variability in other determinants of cost was rarely included.
- Potential determinants of cost that could be included in flexible cost functions for ART provision when modelling over several decades into the future include patient health status and treatment regimen, factor prices, programme/facility scale, facility experience, facility type, quality of care, and the technical efficiency of staff.
- A worked example of a flexible cost function modelling the impact of one of these determinants, programme scale, on the costs of a proposed universal testing and treatment programme in South Africa found that the inefficiencies of small scale could add up to 42% to the total future cost of the programme.
- Another article in this *PLoS Medicine* collection [10] discusses additional operational and effectiveness issues relevant for the economic evaluation of scaling up ART for prevention.

## Supporting Information

Text S1 **Methods and results of previously published modelled economic analyses.**
(PDF)

Text S2 **The algebra of flexible cost functions.**
(PDF)

## Abbreviations

ART: antiretroviral therapy
LMIC: low- and middle-income country
NGO: non-governmental organisation
