# Supplementary material for: HIV Treatment as Prevention: Modelling the Cost of Antiretroviral Treatment—State of the Art and Future Directions
Source: PLoS Med. 2012 Jul 10;9(7):e1001247. doi: 10.1371/journal.pmed.1001247 (PMC3393674; doi:10.1371/journal.pmed.1001247)
Supplement: Text S1 — Methods and results of previously published modelled economic analyses. (PDF) [file pmed.1001247.s001.pdf]

Text S1: Methods and results of previously published modelled economic analyses

Supporting information for

HIV Treatment as Prevention: Modelling the Cost of Antiretroviral Treatment- State of the Art and Future Directions

Gesine Meyer-Rath[1,2] and Mead Over[3]

[1] Center for Global Health and Development, Boston University, Boston, US
[2] Health Economics and Epidemiology Research Office (HE^{2}RO), Department of Medicine, Faculty of Health Sciences, University of the Witwatersrand, Johannesburg, South Africa
[3] Center for Global Development, Washington DC, US

Types of articles identified

Starting in 1992, a wealth of papers have been published on the economics of antiretroviral treatment. The first papers were prompted by the need to make the economic case for public-sector provision and funding of ART in high-income countries, pointing to the beneficial effect of ART not only on survival and quality of life but also on shifting resources from expensive inpatient care to cheaper outpatient care and from the treatment to the prevention of opportunistic infections. From about 2001 on, the same methods were used to also make the case for extending ART provision to low- and middle income countries (LMIC) characterised by both higher HIV prevalence and lower ability to pay for the programmes themselves.

Publications included two modelled cost analyses for two high-income countries and 23 modelled cost-effectiveness or -utility analyses for nine high-income countries (HIC) as well as 13 cost-effectiveness or -utility analyses for six low- and middle income countries. Five of these analyses specifically looked included the impact of ART on HIV transmission in one high- and two middle-income countries; since the focus of this collection is on the impact of ART on HIV transmission, we summarised these separately. We also found four analyses of regional cost and cost-effectiveness of ART and eight studies of the global cost and cost benefit of ART, either for all countries world-wide or for a large number of LMIC. None of these regional and global analyses included an impact on transmission. Table S1 summarises the number of papers and methods used in estimating input costs as well as the results in each category.

Methods used in previously published modelled economic analyses of ART

Single country analyses without transmission impact

We identified 33 modelled economic analyses of single-country ART programmes[1-33]. Most of the 24 HIC analyses compared the incremental cost and effectiveness of a drug regimen of one phase of antiretroviral drug development to that of one of the former, with the biggest output of such analyses being prompted by the introduction of new classes of drugs such as protease inhibitors[7,9-11,18,21,23] and a fusion inhibitor[22-24]. Apart from four studies adopting a societal perspective[5,13,16,17] (only one of which specifically including indirect costs[5]), all analyses analysed cost from a provider perspective, with some specifically identifying the payers and comparing different cost reimbursement strategies[12,19] or the impact of earlier treatment initiation[1,12,15,16]. Amongst the nine LMIC analyses, six analyses focussed on the choice of eligibility criteria[25-27,30,30,33], with two analyses prompted by the revised World Health Organization (WHO) treatment guidelines issued in late 2009[30,31]. One analysis compared ART with no ART[28], one first-line treatment with first- and second line treatment[29], and one different regimens for women previously exposed to single-dose nevirapine as part of PMTCT[32].

The source of cost data for all single-country analyses were real world settings- trial data for most HIC analyses, single-site clinic cohorts for most LMIC settings. Data for drug costs often came from national formularies, using average wholesale prices, or, for studies in LMIC, from drug price databases maintained by WHO (Global Price Reporting

Mechanism), the Clinton Health Access Initiative (CHAI), or the Global Fund for AIDS, Tuberculosis and Malaria (GFATM). Inpatient costs and resource utilisation were distilled from databases or insurance reports or from data maintained by WHO's ChoICE team. Data on laboratory costs came from individual hospitals' payment offices or from previously published studies. Costs were discounted in almost all studies at rates between 3% and 6% per annum. The majority of studies in LMIC used a 3% discount rate. Very few studies varied the discount rate in sensitivity analysis[4,11,14].

A majority of the analyses employed health state transition models, mostly using Markov techniques, while seven studies used versions of the same health state transition model evaluated by Monte Carlo simulation, the Cost-Effectiveness of Preventing AIDS Complications (CEPAC) model[15-18] or its international version[26,30,32]. The time horizon of these models, ie, the period over which outcomes and/ or cost were projected forward, was set at between one and 20 years, or analyses, most often in Markov models, were run for the lifetime of the cohort without further specification. Seven of the 33 analyses were run over five or less years[1,6,7,9,11,12,14,27], ten for five to 25 years[3,4,7,9,10,11,14,25,20,29], and 15 for the lifetime[2,5,8,15-19,24,26,28,30-32] or the half life of the cohort[21]. Three models projected for two different time horizons[7,9,24]; four analyses did not give information on their time horizons[13,20,22,23]. Models further varied according to their assumptions about the duration of a beneficial effect of the ART regimen under study and their output parameter- about half of all analyses used cost per life-year saved (where the difference in average per patient cost between the comparator arms is divided by the difference in average survival) and the other half used cost per quality-adjusted life-year (QALY) gained (where each incremental year of survival is additionally valued by its utility, by multiplying it with a quality of life weight between 0 and 1).

In terms of the use of cost functions, most papers varied input cost (ie, the cost per patient per unit of time) by protocol-related factors such as treatment regimen, health state (defined by the absence and presence of symptoms, opportunistic infections or AIDS-defining diseases and/ or CD4 cell count levels) and/ or by time on treatment (see Table 1). Only two papers, both of them on LMIC, varied cost by level of care (secondary vs. tertiary)[28] or mode of healthcare provision (public vs. private)[29]; none of the papers varied per patient cost by scale or other programmatic factors.

Regional analyses without transmission impact

We found four modelled analyses of the cost of ART provision in a specific region[34-37], all of which focussed on sub-Saharan Africa (with one study additionally including South East Asia[37]). Studies modelled the cost of defined increases in ART coverage from a low baseline[34,35] and the cost effectiveness of ART provision through the specific setting of an antenatal care clinic[36]. Details with regards to model characteristics or sources of input data were unavailable for two analyses[34,35]; one publication was a systematic review of cost-effectiveness analyses of HIV interventions, with the cost of ART modelled on the cheapest available prices at the time[36]; the other used an epidemiological model[37]. All analyses were conducted from the provider perspective. Time horizons, where available, were five years[34], eight years[37], and lifetime[37].

One paper used the same constant input cost for all patients[37]; two papers varied input cost by regimen [34,37]. None of the papers varied per patient cost by any other factors.

Global analyses without transmission impact

Eight papers estimated the cost of global antiretroviral treatment provision[38-45]. Published between 1997 and 2011, they describe a clear evolution in both data availability and modelling technique. Almost all papers analyse the global cost of ART provision only, with the exception of one paper modelling the incremental cost effectiveness of UNAIDS' new "investment approach" to achieving universal ART access[45] and one paper analysing the cost benefit of maintaining the 2011 cohort of patients supported by the Global Fund to AIDS, Tuberculosis and Malaria [44]. While the older analyses estimate cost only based on the number of HIV positive people from a number of sources, varying assumptions of start coverage, and cost modelled on both guidelines and prices from high-income countries[38-40], later analyses model global cost under concrete programmes, such as WHO's "3 by 5" programme[42] and the GFATM[41,43], based on per-

patient cost estimates from relevant low- and middle income countries and more advanced epidemiological models of the number of patients in need of ART, such as the Spectrum model[43,44] and the Resource Needs Model[45]. Accordingly, all analyses are conducted from the provider perspective, with the exception of the cost-benefit analysis which adopted a societal perspective[44]. Time horizons vary between one and ten years.

Three of the eight analyses use constant input costs for all patients[38-40]; two vary input cost by regimen[42,43], and one additionally by health state[43]. One study includes the impact of access to pool procurement prices negotiated by the Clinton HIV/AIDS Initiative on per patient cost[42], one varies drug prices by per capita Gross National Product[41] and one assumes a reduction of per patient cost by 65% by 2020 as a result of task-shifting and cheaper point-of-care diagnostics[45]. No other cost factors are considered.

Single country analyses with transmission impact

Five publications between 2006 and 2011 have analysed the cost of ART for a single country including an impact of treatment on HIV transmission and hence, the number of future infections and future cost[46-50]. Three of these analyses are cost-effectiveness analyses of different strategies of eligibility and coverage[46-48]; two are analyses of the cost impact and cost benefit of earlier treatment initiation, including universal testing and treatment[49,50]. All five analyses use health-state transition models with long time horizons of 20[46], 30[50] and 43[49] years or lifetime[47,48]. Two analyses use the societal perspective[46,48], three a provider perspective[47,49,50].

Three of the analyses vary input cost by regimen[48-50], three by health state[46-48], and one by time on treatment[47]; additionally one analysis varies input cost by whether treatment is administered in a structured way in the public sector or an unstructured way in the private sector[57]. No other variation in cost was considered.

Results of previously published modelled economic analyses of ART

The results of all reviewed papers are summarised in Table 1. As can be seen, the ranges even for the same outcome parameter (life years saved, QALYs gained, or total annual cost) are wide as a result in the variation in methods discussed above, whether a concentrated or a generalised epidemic was studied, and the evolution in the availability of appropriate local cost data. In summary, cost was higher when ART was started earlier and maintained for longer, second line drugs were included in the analysis, and transmission effects were excluded.

Table S1: Economic analyses for single countries (no transmission impact assumed)

First author, year	Country	Aim and intervention(s)	Modelling method; time horizon	Perspective	Measure	Result in 2011 USD	Factors influencing input cost (including in sensitivity analysis, *SA*)[1]
Oddone 1993 (1)	US	Incremental cost effectiveness of early (at recruitment) vs. late (at 200 CD4 cells/microl) initiation of ZDV monotherapy (1500 mg vs. 500 mg per day)	Markov; 4 years	Provider	Cost per month without AIDS	$17,944 (1500 mg); $6,538 (500 mg)	Health state;<br><br>SA: ZDV dosage
Schulman 1991 (2)	US	Incremental cost effectiveness of ZDV monotherapy over no treatment	Health state transition; lifetime	Provider	Cost per life year saved	$9,027 (when continuous benefit is assumed) to $84,882 (when one-time benefit is assumed)	Constant cost in main analysis;<br><br>SA: ZDV cost +/- 50%; lifetime cost in AIDS state +/- 50%
Davies 1999 (3)	UK	Incremental cost effectiveness of ZDV+3TC over ZDV alone in 2 different London clinics	Markov; 25 years	Provider	Cost per life year saved	$14,400 to $32,171	Regimen, health state (CD4 200 | 500 cells/microl);<br><br>no SA
Chancellor 1997 (4)	UK	Full and incremental cost effectiveness of ZDV and ZDV+3TC	Markov; 20 years	Provider	Cost per life year saved	$13,781 (ZDV, full)<br>$17,330 (3TC incremental over ZDV)	Regimen, health state (CD4 200 | AIDS);<br><br>SA: Community cost included
Mauskopf 1998 (5)	US	Incremental cost effectiveness of 3TC+ZDV over ZDV alone	Markov; lifetime	Provider	Cost per life year saved / per QALY	$14,918 to $26,852/<br>$20,885 to $40,279	Regimen, health state (CD4 100 | 200 | 350 | 500);<br>SA: Cost not included
Simpson 1994 (6)	France, Germany, Italy, Switzerland, UK	Incremental cost effectiveness of ddC+ZDV over ZDV alone	Markov; 1 year	Provider	Cost per life year saved	$27,741 (France), $37,154 (Germany), $25,275 (Italy), $31,374 (Switzerland), $42,944 (UK)	Regimen, incidence of opportunistic infections (OI) and AIDS-defining disease (ADD) by CD4 (no details on CD4 categories);<br>SA: Future cost +/- 50%, OI/ ADD incidence +/- 50%
Biddle 2000 (7)	France, Germany, Italy, Spain, US	Incremental cost effectiveness of NVP-containing triple therapy over dual therapy	Markov (based on Simpson 1994 (6) and Chancellor 1997 (4)); 1 year/ 15 years	Provider and patient	Cost per life year saved	$24,509 (France), $25,070 (Germany), $23,328 (Italy), $12,507 (Spain), $20,376 (US)	1 year analysis: same as Simpson 1994 (6)<br>15-year analysis: modified from Chancellor 1997 (4): Regimen, health state (CD4 200 | 500 | AIDS);<br><br>SA: Admission rates in Italy set to be the same as in Spain
Sendi 1999 (8)	Switzerland	Incremental cost effectiveness of HAART over non-HAART	Markov; lifetime	1. Provider,<br>2. Societal	Cost per life year saved	1. (provider perspective):<br>$71,111 (pessimistic scenario),<br>$42,149 (base case),<br>$22,124 (optimistic scenario)<br>2. (societal perspective):<br>$17,383 (pessimistic scenario),<br>cost savings in base case and optimistic scenario	Health state (CD4 200 | 500, both with and without AIDS);<br><br>SA: 95% confidence intervals around all estimates (probabilistic SA)

[1] For health states, the notation "CD4 200 | 350" denotes the cut-off values between CD4 cell count categories; the corresponding categories would be <200, 200-350, and >350 cells/microl

First author, year	Country	Aim and intervention(s)	Modelling method; time horizon	Perspective	Measure	Result in 2011 USD	Factors influencing input cost (including in sensitivity analysis, *SA*)[1]
Cook 1999 (9)	US	Incremental cost effectiveness of ZDV+3TC+IDV over ZDV+3TC	Health state transition with semi-Markov model; 5/ 20 years	Provider	Cost per life year saved	$19,174	Regimen, health state (CD4 200 | 500 | AIDS); ART given until VL returns to baseline;<br>SA: Different set of cost estimates (but same CD4 categories); ART given until time of index ADD or death
Trueman 2000 (10)	UK	Incremental cost effectiveness of triple over dual NRTI therapy	Markov (same as Chancellor 1997 (4)); 20 years	Provider	Cost per life year saved/ per QALY	$17,217/<br>$20,598 (optimistic scenario),<br>$33,064 (pessimistic scenario)	Regimen, health state (CD4 200 | AIDS);<br><br>SA: Time horizon 5 years only
Miners 2001 (11)	UK	Incremental cost effectiveness of HAART over dual NRTI	Markov; 20 years	Provider	Cost per life year saved/ per QALY	$35,897/ $43,508	Regimen, health state (CD4 200 | AIDS) and time on treatment (first year vs consecutive years);<br>SA: Increase in cost of third drug; time horizon 10 years
Kahn 2001 (12)	US	Incremental cost effectiveness of increased access to HAART by expanding Medicaid	Markov; 5 years	Provider	Cost per life year saved with limited benefits package (drugs and outpatient care)	$17,383	Health state (CD4 200, asymptomatic | 500, asymptomatic | symptomatic, pre-AIDS | AIDS (1993 definition) | AIDS (1987 definition)); medication payor; full vs. limited benefit paid<br>SA: Cost of ART +/- 20%; cost of all other medical care +/- 40%; insurance mix; eligibility expansion
Risebrough 1999 (13)	Canada	Incremental cost benefit of IDV+ZDV+3TC and ABC+ZDV+3TC over ZDV+3TC	Markov; n.a.	Society	Cost per life year saved	$54,589 (IDV+ZDV+3TC), $4,389 to $27,516 (ABC+ZDV+3TC, depending on salvage regimen used)	Regimen (HAART vs. salvage therapy), health state (200 | AIDS);<br><br>SA: n.a.
Caro 2001 (14)	US	Cost and effectiveness of EFV- or IDV-containing HAART regimens	Monte Carlo simulation; 5 and 15 years	Provider	Daily cost of EFZ and IDV; mortality rate and progression to AIDS after 5 years	$14.71 (EFV), $20.72 (IDV);<br>11% less mortality and 1,9% less progression to AIDS with EFV over IDV	Regimen (two 1st line, one 2nd line, salvage therapy), health state ("responsive, tolerant and willing to adhere" | treatment failure | AIDS | final year);<br>SA: Treatment cost 10-200% (EFV-containing regimen), 50-300% (IDV-containing regimen)
Schackman 2002 (15)	US	Full cost effectiveness of early initiation of HAART (i.e., at ≤ 350 vs. ≤ 200 CD4 cells/microl) in patients with low viral load	Health state transition with Monte Carlo simulation (CEPAC model); lifetime	Provider	Cost per QALY gained	$16,430 (early initiation without QoL adjustment for fat redistribution syndrome), $21,485 to $295,113 (with QoL adjustment for fat redistribution syndrome)	Regimen (1st, 2nd, 3rd and 4th line) and incidence of OIs and ADDs by health state (CD4 50 | 100 | 200 | 300 | 500);<br><br>no SA

First author, year	Country	Aim and intervention(s)	Modelling method; time horizon	Perspective	Measure	Result in 2011 USD	Factors influencing input cost (including in sensitivity analysis, *SA*)[2]
Schackman 2001 (16)	US	Incremental cost effectiveness and state budget impact of early (i.e., at CD4 ≤ 500 cells/µl) and late (i.e., at CD4 ≤ 200 cells/µl) initiation of HAART over no therapy	CEPAC model; lifetime(?)	Society	Cost per QALY gained	$22,839 (early), $26,403 (late)	One triple therapy regimen only; health state (CD4 50 | 100 | 200 | 300 | 500); acute OI episodes (not by health state); US state (MA/ NY/ FL/ national average);<br><br>SA: Additional 3rd and 4th line; drug prices +/- 50%
Yazdanpanah 2002 (17)	France	Lifetime cost and cost by clinical stage	CEPAC model; lifetime	Society	Lifetime cost; cost per pt month	Lifetime cost $310,345;<br>cost per pt month from $739 (CD4>500) to $11,090 (final month before death)	Regimen (1st, 2nd, 3rd , and 4th line) and health state (no history of or current AIDS, by CD4 cell count | current AIDS | history of ADD but currently no AIDS | final month of life);<br>SA: Dosage of ARV drugs (+/-25% and +/-50%), duration of outpatient medication usage (50%, 75%, 90%), four consecutive lines of very efficacious/ low efficacy ART
Freedberg 2001 (18)	US	Incremental cost effectiveness of HAART using data from 4 different cohorts (ACTG, JH, INCAS, Dupont)	CEPAC model; lifetime(?)	Provider	Cost per QALY gained	$32,076 (ACTG), $23,708 (JH), $18,129 (INCAS and Dupont)	Regimen (1st/ 2nd line) and health state (CD4 50 | 100 | 200 | 300 | 500 and VL 500 | 3000 | 10,000 | 30,000 cop/ml);<br>SA: Drug prices +/- 50%; OI treatment and routine care cost +/- 50%
Mauskopf 2000 (19)	US	Incremental cost to medical system of treating 100 pts under the AIDS Drug Assistance Program (ADAP)	Static deterministic health state model; lifetime	Provider(?)	Incremental cost	Incremental ADAP cost for HAART to 100 pts.: $924,383<br>Decrease in total medical care cost, including drugs, for 100 pts.: $9,914	Health state (CD4 100 | 200 | 350 | 500);<br><br>SA: Drug cost +/- 10%; OI event cost +10% and +/- 25%
Moore 1996 (20)	US	Incremental cost effectiveness of 3TC+IDV+ZDV over ZDV alone	Health state transition; n.a.	Provider	Cost per life year saved	$16,201 to $29,162 (depending on the increase in other health care cost)	Regimen; health state (CD4 200 | 500 | AIDS);<br>no SA
Simpson 2004 (21)	US	Incremental cost effectiveness of LPV/r+d4T+3TC over NFV+d4T+3TC as first line regimen	Markov model; run until 50% of pts had died	Provider	Cost per life year saved/ per QALY gained	$8,058/ $8,408 (not taking resistance development into account), cost savings (taking resistance into account)	Regimen, health state (CD4 50 | 200 | 350 | 500 and VL 400 | 20,000 | 100,000 cop/ml) and) and incidence of OIs and ADDs by health state;<br>SA: Cost of OI events by 50-200%; cost of LPV/r
Munakata 2003 (22)	Canada	Incremental cost effectiveness of adding enfuvirtide to an (unspecified) ART background regimen for treatment-experienced pts	Markov model; n.a.	Provider	Cost per life year saved/ per QALY gained	$178,915/<br>$248,189	Regimen; no other information available;<br><br>no SA

[2] For health states, the notation "CD4 200 | 350" denotes the cut-off values between CD4 cell count categories; the corresponding categories would be <200, 200-350, and >350 cells/microl

First author, year	Country	Aim and intervention(s)	Modelling method; time horizon	Perspective	Measure	Result in 2011 USD	Factors influencing input cost (including in sensitivity analysis, *SA*)[3]
Snedecor 2005 (23)	US	Incremental cost effectiveness of HAART over non-HAART and of unspecified 'rescue regimen with 10% greater efficacy' over HAART	Monte Carlo Markov model; n.a.	Provider	Cost per QALY gained	HAART: $27,164<br>rescue regimen: $16,029	Regimen (two 1st line regimens, one rescue regimen) and health state (CD4 categories n.a.);<br><br>no SA
Sax 2005 (24)	US	Incremental cost effectiveness of a 4-drug regimen (2 PI+2 NRTI) plus enfuvirtide (ENF) over 4-drug regimen alone	Health state transition with Monte Carlo simulation; lifetime	Provider	Cost per QALY gained	$89,229 (if ENF is administered only until VL returns to pre-treatment level); $215,947 (if ENF is given until death)	Regimen and health state (CD4 50 | 100 | 200 | 300 | 500 and VL 500 | 3000 | 10,000 | 30,000 | 100,000 cop/ml); ENF given until VL returns to baseline;<br>SA: ENF cost (50-200%), continuation of ENF until death
Long 2006 (25)	Russia	Effectiveness and cost-effectiveness of providing HAART to HIV+ IDUs and non-IDUs in Russia, comparing providing HAART only to IDUs (IDU-targeted strategy), only to non-IDUs (non-IDU targeted strategy), or to all HIV+ patients regardless of IDU status (untargeted strategy)	Dynamic compartmental model; 20 years	n.s.	Cost per QALY gained over next best strategy, infections averted<br><br>20 yr time horizon	*IDU targeted strategy*: incremental cost effectiveness over non-IDU targeted programme $1,682 per QALY gained<br><br>*Non-IDU targeted strategy:* incremental cost effectiveness over current program $2,883 per QALY gained<br><br>*Untargeted strategy:* incremental cost effectiveness over IDU targeted strategy $2,104 per QALY gained<br><br>*Optimistic untargeted strategy:* incremental cost effectiveness over untargeted strategy $2,048 per QALY gained	Constant cost;<br><br>SA: Variation on ART and counselling cost
Goldie 2006 (26)	Cote d'Ivoire	Incremental cost effectiveness of 22 different starting and treatment options in ARNS trial cohort	Health state transition with Monte Carlo simulation; lifetime(?)	Modified societal (patients' time and travel cost excluded)	Incremental cost per life year gained for a) cotrimoxazole prophylaxis,<br>b) for ART and cotrimoxazole without CD4 testing,<br>c) for ART and cotrimoxazole with CD4 testing	a) US$ 295,<br>b) US$ 761,<br>c) US$ 1,449	Only 1st line in main analysis (2nd line in SA); health state (CD4 200 | terminal care); OI incidence dependent on CD4 and history of previous OI;<br><br>SA: Additional 2nd line

[3] For health states, the notation "CD4 200 | 350" denotes the cut-off values between CD4 cell count categories; in this case, the corresponding categories would be <200, 200-350, and >350 cells/microl

First author, year	Country	Aim and intervention(s)	Modelling method; time horizon	Perspective	Measure	Result in 2011 USD	Factors influencing input cost (including in sensitivity analysis, *SA*)[4]
Paton 2006 (27)	Singapore	Cost and cost-effectiveness of ART for HIV based on CDC stage of HIV infection (1. dual ART and 2. HAART)	n.a.; 5 years	Provider	Incremental cost per life year gained	*CDC stage A :*<br>1. $11,247; 2. $14,886<br>*CDC stage B :*<br>1. $7,187; 2. $13,949<br>*CDC stage C:*<br>1. $6,512; 2. $10,920	
Cleary 2006 (28)	South Africa	Cost and incremental cost-effectiveness of ART over no ART treatment	Markov model; lifetime	Provider	Total (incremental) cost per patient year/ per QALY gained<br><br>a) ART compared to No ART<br><br>b) Initiating ART when CD4<50 compared to starting when CD4 50-199	Cost per patient year:<br>a) $14,901 and $13,203<br>b) $15,018 and $14,781<br><br>Cost per QALY gained:<br>a) $18,280 and $18,851<br>b) n/a<br><br>Incremental cost per QALY gained:<br>a) $18,106<br>b) $12,722	Regimen (1st line, 2nd line) and, for the first 6 months on ART, health state (CD4 50 | 200), time on ART (3-monthly until 6 months on ART, 6-monthly until 36 months), inpatient cost by type of hospital (secondary vs. tertiary);<br><br>SA: 95% confidence intervals for all results (probabilistic SA)
Over 2007 (29)	Thailand	Cost effectiveness of Thailand's National Access to Antiretroviral Program for People Living with HIV/AIDS (NAPHA) programme	Deterministic difference-equation model with conditional demand allocation for different treatment modes; 20 years	Provider	Cost per life year saved	*First-line drugs only:*<br>$868 per LY saved<br><br>*First- and second-line drugs:*<br>- currently: $2,540 per LY saved<br>- after issuing compulsory licenses (leading to a 90% reduction in the future cost of second-line drugs): $1,108 per LY saved	All cost (including inpatient and outpatient service cost, not only drug cost!) by regimen (drug costs as weighted averages of six 1st line regimens and two 2nd line regimens, resp.); health state (asymptomatic | symptomatic), and mode of service delivery (public vs. augmented public vs. private)<br><br>Other scenarios considered: Compulsory licensing for 2nd line drugs
Walensky 2010 (30)	South Africa	Incremental cost effectiveness of implementing elements of the 2010 WHO guidelines:<br>1. Routine CD4 monitoring<br>2. d4T- vs. TDF-based first line<br>3. Initiation by WHO stage vs. at <200 CD4 cells/microl vs. at <350 CD4 cells/microl<br>4. First-line only vs. first- and second-line ART	CEPAC-International model; lifetime(?)	n.a.	Cost per life year saved	Three "economically efficient" combinations:<br>- *Stavudine/ <350/ml/ one line*: $614/ YL saved<br>- *Tenofovir/ <350/ml/ one line*: $1,197/ YL saved<br>- *Tenofovir/ <350/ml/ two lines*: $2,489/ YL saved	Regimen (two 1st line, one 2nd line), health state (CD4 50 | 100 | 200 | 300 | 500 and VL 500 | 3000 | 10,000 | 30,000 cop/ml);<br><br>SA: Cost of TDF, 2nd line and CD4 cell count tests

[4] For health states, the notation "CD4 200 | 350" denotes the cut-off values between CD4 cell count categories; in this case, the corresponding categories would be <200, 200-350, and >350 cells/microl

First author, year	Country	Aim and intervention(s)	Modelling method; time horizon	Perspective	Measure	Result in 2011 USD	Factors influencing input cost (including in sensitivity analysis, *SA*)[5]
Bendavid 2011 (31)	South Africa	Incremental cost-effectiveness of different first-line regimens:<br>1.TDF/3TC/NVP<br>2. TDF/3TC/EFV<br>3. AZT/3TC/NVP<br>4. AZT/3TC/EFV<br>5. d4T/3TC/NVP	Simulation model; lifetime	Societal	Cost per QALY gained	1. Base<br>2. Dominated<br>3. $1,098 per QALY gained<br>4. Dominated<br>5. $6,250 per QALY gained	Regimen (five 1st line , one 2nd line), health state (200 | 350)<br><br>SA: probabilistic
Ciaranello 2011 (32)	South Africa	Incremental cost effectiveness of<br>1.no ART<br>2. LPV/r-based ART<br>3. NVP-based ART<br>in women after sdNVP exposure for PMTCT	CEPAC-International model; lifetime(?)	Modified societal	Life years saved, cost and ICERs	1. 1.6 yrs; $3,130<br>2. $851/LY saved (vs. 1)<br>3. $1,597/LY saved (vs. 2)	Regimen (4 regimens and "3rd line maintenance" regimen) and health state (200 | terminal care)<br><br>SA: Frequency of VL monitoring, additional 3rd line regimen
Bachmann 2006 (33)	South Africa	Incremental cost effectiveness of early (CD4<350) and late (CD4<200) prevention of progression of HIV/AIDS with ART or antibiotics	Markov Monte Carlo simulation; 10 years	Provider	Cost per QALY gained	Early intervention:<br>ART only $3,345<br>ART+ antibiotics $15,324<br>Antibiotics $295<br><br>Late intervention:<br>ART only $2,983<br>ART+ antibiotics $3,024<br>Antibiotics only $21	Time on treatment (first 3 months vs. thereafter) and health state (tuberculosis | other infection | no infection, at below or above CD4 200);<br><br>no SA

3TC: lamivudine; ABC: abacavir; AZT: zidovudine; d4T: stavudine; ChoICE: WHO's "CHOosing Interventions that are Cost-Effective" Team; DALY: disability-adjusted life-year; ddC: zalcitabine; EFV: efavirenz; GFATM: Global Fund to fight AIDS, Tuberculosis and Malaria, HAART: highly-active antiretroviral therapy; ICER: incremental cost-effectiveness ratio; IDV: indinavir; LPV/r: lopinavir/ ritonavir; LY: life years; n.a.: not available; NRTI: nucleoside reverse transcriptase inhibitor; NVP: nevirapine; PMTCT: prevention of mother-to-child transmission; pt: patient; pts: patients; QALY: quality-adjusted life-year; QoL: quality of life; SA: sensitivity analysis; TDF: tenofovir; USD: US dollar; VL: viral load; WHO: World Health Organization; yr: year; ZDV: zidovudine

[5] For health states, the notation "CD4 200 | 350" denotes the cut-off values between CD4 cell count categories; in this case, the corresponding categories would be <200, 200-350, and >350 cells/microl

Table 2: Regional economic analyses (no transmission impact assumed)

First author, year	Region	Aim and method	Modelling method; time horizon	Perspective	Measure	Result in 2011 USD	Factors influencing input cost (including in sensitivity analysis, *SA*)
Bonnel 2000 (34)	Sub-Saharan Africa	Cost of scaling up ART by 10% in countries with very low and low current HIV programme strength, and by 25% in countries with a medium or strong current HIV programme	n.a., 5 years	Provider	Cost per patient year<br><br>Total annual cost	$2,993 - $5,208<br><br>$2.3 - 3.6 billion	Regimen (drug costs set at 73%-86% of current US drug prices);<br><br>no SA
Kumaranayake 2001 (35)	Sub-Saharan Africa	Incremental cost of ART provision (target coverage of 48% in 2007 and 62% in 2015)	n.a.; 8 years	Provider	Total annual cost	$4.0 to 6.5 billion (2007);<br>$5.8 to 9.3 billion (2015)	No details available, but cost likely to be constant;<br><br>no SA
Creese 2002 (36)	Sub-Saharan Africa	Incremental cost-effectiveness of ART based on previously published estimates	Systematic review ; n.a.	Provider	Cost per life year gained	$1,582 -2,608	Constant cost;<br><br>no SA
Hogan 2005 (37)	Sub-Saharan Africa and South East Asia	Cost effectiveness of ART provided through antenatal care clinics	Epidemiological model; lifetime(?)	n.a.	1) Cost per infection averted<br><br>2) Cost per DALY averted	*No intensive monitoring, 1st line drugs:*<br>1) $42,109 2) $835<br>*Intensive monitoring, 1st line drugs:*<br>1) $52,302 2) $895<br>*No intensive monitoring, 2nd line drugs:*<br>1) 271,985 2) $3,019<br>*Intensive monitoring, 2nd line drugs:*<br>1) $278,436 2) $2,969	Regimen (1st line, 2nd line), type of monitoring;<br><br>SA: Variation of programme cost in relation to patient cost

3TC: lamivudine; ABC: abacavir; AZT: zidovudine; d4T: stavudine; ChoICE: WHO's "CHOosing Interventions that are Cost-Effective" Team; DALY: disability-adjusted life-year; ddC: zalcitabine; EFV: efavirenz; GFATM: Global Fund to fight AIDS, Tuberculosis and Malaria, HAART: highly-active antiretroviral therapy; ICER: incremental cost-effectiveness ratio; IDV: indinavir; LPV/r: lopinavir/ ritonavir; LY: life years; n.a.: not available; NRTI: nucleoside reverse transcriptase inhibitor; NVP: nevirapine; PMTCT: prevention of mother-to-child transmission; pt: patient; pts: patients; QALY: quality-adjusted life-year; QoL: quality of life; SA: sensitivity analysis; TDF: tenofovir; USD: US dollar; VL: viral load; WHO: World Health Organization; yr: year; ZDV: zidovudine

Table 3: Global economic analyses (no transmission impact assumed)

First author, year	Countries/ Regions	Aim and method	Modelling method; time horizon	Perspective	Measure	Result in 2011 USD	Factors influencing input cost (including in sensitivity analysis, *SA*)
Floyd 1997 (38)	Worldwide	Cost of global ART provision (100% coverage)	Estimation based on population and prevalence data; n.s.	Provider	Cost per patient year<br><br>Total annual cost	- AZT monotherapy: $6,252 to $8,269<br>- triple ART (excluding ritonavir): $15,368 to $24,344<br><br>-Triple ART: $133.3 - $176 billion globally (Sub-Saharan Africa: $74.5 - $98.4 billion, Southeast Asia $41.7 - $55 billion, Latin America $6.6 - $8.8 billion, North America $5.9 - $7.9 billion, Western Europe $4.5 - $5.9 billion)	Constant cost data using drug prices from US, laboratory and hospital cost data from US, Uganda, South Africa and Malawi, resource use modelled on UK guidelines;<br><br>no SA
Hogg 1998 (39)	Worldwide	Cost of global ART provision (25% coverage)	Estimation based on population and prevalence data and coverage in British Columbia; 1 year	Provider	Total annual cost	$110 billion globally (95% CI 35 - 189 billion (Sub-Saharan Africa $75 billion, South and South East Asia $22 billion, Americas $8 billion, Western Europe $1.7 million)	Constant drug cost using data from the US;<br><br>SA: Drug cost reduced by 50, 75, 90 and 99%; additional probabilistic analysis
Attaran 2001 (40)	Worldwide	Cost of global ART and prevention	Estimation based on prevalence data and assumed cost of ART; 3 years	Provider	Total annual cost	$10.8 billion	Constant assumed cost of ART and palliative care $500, and of prevention $10 per pt yr;<br><br>no SA
Schwartländer 2001 (41)	135 low- and middle-income countries	Cost of global ARV drugs and laboratory monitoring for eligible patients	Model based on UNAIDS estimates of population in need, access to care assumptions, 5 years	Provider	Cost per patient year in 2005<br><br>Total annual cost	$826-5,467<br><br>$3.8 billion (27% of total resource need for treatment and prevention)	Per-capita Gross National Product (differential pricing for drugs), age (cost of care for children assumed to cost 50% of adult care);<br><br>no SA
Guiterrez 2004 (42)	Worldwide	Cost of 3 by 5 programme (ART to 3 million eligible patients by 2005)	Health-state transition model; 2 years	Provider	Total cost of programme	$6.4 - 7.4 billion	Regimen (two 1st line, one 2nd line), current prices or prices negotiated by Clinton Foundation;<br><br>no SA
Stover 2011 (43)	104 low- and middle income countries receiving support from GFATM	Cost of maintaining 3.5 million people currently supported (with 25% of total cost) by GFATM on ART in 2011-2020	Spectrum model; 10 years	Provider	Annual cost of ART to 2011 GFATM cohort<br><br>Life-years saved per year	$2 billion (2011), $1.8 billion (2020)<br><br>830,000 (2011), 2.3 million (2015-2020)	Regimen (1st line, 2nd line); end-of-life treatment separately<br><br>SA: Reduction in ARV drug prices per year: 5% in 1st line, 11% in 2nd line drugs; replacement of d4T by other drugs; migration to 2nd line 6% per year

First author, year	Countries/ Regions	Aim and method	Modelling method; time horizon	Perspective	Measure	Result in 2011 USD	Factors influencing input cost (including in sensitivity analysis, *SA*)
Resch 2011 (44)	104 low- and middle income countries receiving support from GFATM	Cost benefit of maintaining 3.5 million people currently supported (with 25% of total cost) by GFATM on ART in 2011-2020	Spectrum model; 10 years	Societal	Total programme cost<br><br>Total programme benefit	$14.9 billion<br><br>$13-$36 billion (94% of which due to productivity gains)	Cost based on Stover 2011; benefits:<br>- productivity gains (valued by per-capita income)<br>- orphanhood avoided (cost based on literature)<br>- end of life care postponed (literature)<br><br>SA: Productivity of treated/ untreated patients in relation to asymptomatic patients; valuation of productivity by friction cost only
Schwartländer 2011 (45)	Worldwide	Incremental cost effectiveness of "investment approach" to achieving universal access to HIV prevention, treatment, care and support (including interventions, social and programme 'enablers' and synergies with other development sectors)	Resource Needs Model; 9 years	Provider	Cost per LY saved	Incremental cost-effectiveness ratio $1,077 per life year saved<br>Cost: $22 billion; 12.2 million HIV infections averted; 7.4 million deaths from AIDS averted; 29.4 million life-years gained; "additional investment proposed would be largely offset from savings in treatment costs alone"	Not much information given, but "average cost per patient of antiretroviral therapy is assumed to decline by about 65% between 2011 and 2020, with a large proportion of the cost savings after 2015 coming from an increasing shift to primary care and community-based approaches and cheaper point-of-care diagnostics";<br><br>no SA

3TC: lamivudine; ABC: abacavir; AZT: zidovudine; d4T: stavudine; ChoICE: WHO's "CHOosing Interventions that are Cost-Effective" Team; DALY: disability-adjusted life-year; ddC: zalcitabine; EFV: efavirenz; GFATM: Global Fund to fight AIDS, Tuberculosis and Malaria, HAART: highly-active antiretroviral therapy; ICER: incremental cost-effectiveness ratio; IDV: indinavir; LPV/r: lopinavir/ ritonavir; LY: life years; n.a.: not available; NRTI: nucleoside reverse transcriptase inhibitor; NVP: nevirapine; PMTCT: prevention of mother-to-child transmission; pt: patient; pts: patients; QALY: quality-adjusted life-year; QoL: quality of life; SA: sensitivity analysis; TDF: tenofovir; USD: US dollar; VL: viral load; WHO: World Health Organization; yr: year; ZDV: zidovudine

Table 4: Economic analyses for single countries (transmission impact assumed)

First author, year	Country	Aim and intervention(s)	Modelling method; time horizon	Perspective	Measure	Result in 2011 USD	Factors influencing input cost (including in sensitivity analysis, *SA*)
Long 2010 (46)	US	Incremental cost effectiveness of expanded HIV testing and ART	Dynamic model; 20 years	Societal	Cost per QALY gained<br><br>20 yr horizon; lifetime costs	*One-time screening:* $22,649 per QALY gained<br><br>*Expanding ART coverage to 75% of eligible persons:* $20,542 per QALY gained<br><br>*Combination strategy:* $21,840 per QALY gained	One regimen cost only; health state (untreated asymptomatic | untreated symptomatic | treated symptomatic | untreated AIDS | treated AIDS)<br><br>SA: Cost not included
Over 2004 (47)	India	Cost-effectiveness of national ART programme 2003 to 2033<br>- for 40% of eligible pts falling under the poverty line ("Below the Poverty Line")<br>- for 25% of mothers and 1.5% of fathers of children eligible for PMTCT ("MTCT+")<br>- capacity building and subsidies for laboratory tests, with antiretroviral treatment paid for by patients ("ADHERE")	Epidemiologic al model; lifetime(?)	Provider	Cost per life year saved	*Below the Poverty Line*:<br>- no change in condom uptake: $378 per LY saved<br>- 70% condom use rate: $69 per LY saved<br>- 90% condom use rate: $40 per LY saved<br><br>*MTCT+:*<br>- no change in condom uptake: $268 per LY saved<br><br>*ADHERE:*<br>- no change in condom uptake: $197 per LY saved	Time on treatment (first 3 years vs. year before death); health state (symptomatic, non-AIDS | AIDS); unstructured vs. structured treatment provision<br><br>SA: Cost not included
Vijayaraghavan 2006 (48)	South Africa	Incremental cost effectiveness of implementing DHHS treatment guidelines (initiate treatment at CD4<350 or viral load>100,000 and monitor with CD4 counts and viral load every three months) over WHO guidelines (initiate treatment at CD4<200 or for patients with AIDS and monitor using CD4 counts every 6 months)	Markov model with Monte Carlo simulation; lifetime	Societal	Incremental cost per QALY gained<br>a) not including impact on transmission<br>b) including impact on transmission<br>c) including indirect costs (without transmission)	a) $5,865 per QALY gained<br>b) $4,594 per QALY gained<br>c) $1,550 per QALY gained<br><br>'Over a five-year period, treating all HIV patients in South Africa according to US DHHS versus WHO guidelines would increase direct medical costs by US$14.5 billion but would result in approximately 400,000 fewer deaths and 1.1 million fewer new AIDS cases.'	Regimen (1st line, 2nd line) and health state (if not on ART: CD4 350 | 200 and asymptomatic | symptomatic | AIDS; if on ART, additionally: unsuppressed | toxicity | suppressed | without additional treatment options)<br><br>SA: Cost of VL and of 2nd line +/- 25%,
Granich 2009 (49)	South Africa	Impact of universal voluntary testing and immediate treatment (UTT) on annual cost, HIV incidence and prevalence	Deterministic transmission model and stochastic survival model; 43 years	Provider(?)	Impact on incidence, prevalence, and overall programme cost	Incidence: reduction to <1/1000 per year by 2016 (within 10 yrs of full implementation of UTT)<br><br>Prevalence: reduction to less than 1% within 50 years<br><br>Cost: same as base case until 2032 (US$1.7 billion); lower thereafter	Regimen (1st line, 2nd line);<br><br>no SA

Gesine Meyer-Rath and Mead Over

First author, year	Country	Aim and intervention(s)	Modelling method; time horizon	Perspective	Measure	Result in 2011 USD	Factors influencing input cost (including in sensitivity analysis, *SA*)
Hontelez 2011 (50)	South Africa	Incremental cost benefit of ART initiation at different CD4 cell count thresholds (<200 vs. <350)	Simulation model; 30 years	Provider	Total cost of ART programme	Initiation at <350 costs 7% more per annum during first 5 years, with cost decreases due to reduction in incidence and ART need after 7 years; break-even in cost after on average 16 years	Regimen (1st line, 2nd line), baseline (*not* current) CD4 cell count (100 | 200 | 350) for first three years;<br><br>SA: Cost varied by +/- 33%

3TC: lamivudine; ABC: abacavir; AZT: zidovudine; d4T: stavudine; ChoICE: WHO's "CHOosing Interventions that are Cost-Effective" Team; DALY: disability-adjusted life-year; ddC: zalcitabine; EFV: efavirenz; GFATM: Global Fund to fight AIDS, Tuberculosis and Malaria, HAART: highly-active antiretroviral therapy; ICER: incremental cost-effectiveness ratio; IDV: indinavir; LPV/r: lopinavir/ ritonavir; LY: life years; n.a.: not available; NRTI: nucleoside reverse transcriptase inhibitor; NVP: nevirapine; PMTCT: prevention of mother-to-child transmission; pt: patient; pts: patients; QALY: quality-adjusted life-year; QoL: quality of life; SA: sensitivity analysis; TDF: tenofovir; USD: US dollar; VL: viral load; WHO: World Health Organization; yr: year; ZDV: zidovudine

References for Text S1

1 Oddone E, Cowper P, Hamilton J, Matchar DB, Hartigan P, et al (1993) Cost effectiveness analysis of early zidovudine treatment of HIV infected patients. Brit Med J 307: 1322-5.

2 Schulman K, Lynne L, Glick H, Eisenberg J (1991) Cost-effectiveness of low-dose zidovudine therapy for asymptomatic patients with human immunodeficiency virus (HIV) infection. Ann Intern Med 114: 798-801.

3 Davies D, Carne C, Camilleri-Ferrante C (1999) Combined antiviral treatment in HIV infection. Is it value for money? Publ Health 113: 315-7.

4 Chancellor JV, Hill AM, Sabin CA, Simpson KN, Youle M (1997) Modelling the cost effectiveness of lamivudine/zidovudine combination therapy in HIV infection. Pharmacoeconomics 12(1): 54-66.

5 Mauskopf J, Lacey L, Kempel A, Simpson K (1998)The cost-effectiveness of treatment with lamivudine and zidovudine compared with zidovudine alone: a comparison of Markov model and trial data estimates. Am J Manag Care 4(7): 1004-12.

6 Simpson K, Hatziandreu EJ, Andersson F, Shakespeare A, Oleksy I et al (1994) Cost effectiveness of antiviral treatment with zalcitabine plus zidovudine for AIDS patients with CD4+ counts less than 300/microliters in 5 European countries. Pharmacoeconomics 6(6): 553-62.

7 Biddle AK, Simpson KN (2000) Modeling the use of triple combination therapy in five countries: nevirapine, zidovudine, and didanosine. Value in Health 3(3): 186-201.

8 Sendi PP, Bucher H, Harr T, Craig BA, Schwietert M (1999) Cost effectiveness of highly active antiretroviral therapy in HIV-infected patients. AIDS 13: 1115-22.

9 Cook J, Dasbach E, Coplan P, Markson L, Yin D, et al (1999) Modeling the long-term outcomes and costs of HIV antiretroviral therapy using HIV RNA levels: applications to a clinical trial. AIDS Res Human Retroviruses 15(6): 499-508.

10 Trueman P, Youle M, Sabin CA, Miners AH, Beck EJ (2000) The cost-effectiveness of triple nucleoside analogue therapy antiretroviral regimens in the treatment of HIV in the United Kingdom. HIV Clin Trials 1(1): 27-35.

11 Miners A, Sabin C, Trueman P, Youle M, Mocroft A, et al (2001) Assessing the cost-effectiveness of highly active antiretroviral therapy for adults with HIV in England. HIV Medicine 2: 52-8.

12 Kahn JG, Haile B, Kates J, Chang S (2001) Health and federal budgetary effects of increasing access to antiretroviral medications for HIV by expanding Medicaid. Am J Public Health 91(9):1464-73.

13 Risebrough N, Oh P, Rachlis A, McMurchy D, Bast M, et al (1999) Economic Evaluation of Triple ART with Indinavir or Abacavir and ZDV+3TC Compared to Dual Therapy ZDV+3TC. 6th Conference on Retroviruses and Opportunistic Infections.

14 Caro J, O'Brien J, Miglaccio-Walle K, Raggio G (2001) Economic analysis of initial HIV treatment: efavirenz- versus indinavir-containing triple therapy. Pharmacoeconomics 19: 95-104.

15 Schackman BR, Freedberg KA, Weinstein MC, Sax PE, Losina E, et al (2002) Cost-effectiveness implications of the timing of antiretroviral therapy in HIV-infected adults. Arch Intern Med 162(21): 2478-86.

16 Schackman BR, Goldie SJ, Weinstein MC, Losina E, Zhang H, et al (2001) Cost-effectiveness of earlier initiation of antiretroviral therapy for uninsured HIV-infected adults. Am J Public Health 91(9): 1456-63.

17 Yazdanpanah Y, Goldie S, Losina E, Weinstein MC, Lebrun T, et al (2002) Lifetime cost of HIV care in France during the era of highly active antiretroviral therapy. Antivir Therapy 7: 257-266.

18 Freedberg KA, Losina E, Weinstein MC, Paltiel D, Cohen C, Seage G, et al (2001) The cost effectiveness of combination antiretroviral therapy for HIV disease. N Engl J Med 344: 824-31.

19 Mauskopf JA, Tolson JM, Simpson KN, Pham SV, Albright J (2000) Impact of zidovudine-based triple combination therapy on an AIDS drug assistance program. JAIDS 23(4): 302-313.

20 Moore RD, Bartlett J (1996) Combination antiretroviral therapy in HIV infection: an economic perspective. Pharmacoeconomics 10: 109-13.

21 Simpson KN, Luo MP, Chumney E, Sun E, Brun S, Ashraf T (2004) Cost-Effectiveness of Lopinavir/Ritonavir Versus Nelfinavir As the First-Line Highly Active Antiretroviral Therapy Regimen for HIV Infection. HIV Clin Trials 5(5): 294-304.

22 Munakata J, Sanders G, Owens D, Bayoumi A (2003) Cost effectiveness of enfuvirtide in the treatment of drug-resistant HIV infection. Med Decis Making 23: 569.

23 Snedecor S, Hartzema A, Schiller K (2005) Cost effectiveness of HIV treatment innovations of greater efficacy than highly active antiretroviral therapy (HAART). Value in Health 8(3): 244.

24 Sax P, Losina E, Weinstein M, Paltiel A, Goldie S, et al (2005) Cost-effectiveness of enfuvirtide in treatment-experienced patients with advanced HIV disease. JAIDS 39(1): 69-77.

25 Long E, Brandeau M, Galvin C, Vinichenko T, Tole S, et al (2006) Effectiveness and cost-effectiveness of strategies to expand antiretroviral therapy in St. Petersburg, Russia. AIDS 20: 2207-15.

26 Goldie S, Yazdanpanah Y, Losina E, Weinstein M, Anglaret X, et al (2006) Cost-effectiveness of HIV treatment in resource-poor settings--the case of Cote d'Ivoire. N Engl J Med 355(11): 1141-53.

27 Paton N, Chapman C, Sangeetha S, Mandalia S, Bellamy R, et al (2006) Cost and cost-effectiveness of antiretroviral therapy for HIV infection in Singapore. Int J STD AIDS 17(10):699-705.

28 Cleary S, McIntyre D, Boulle A (2006) The cost-effectiveness of antiretroviral treatment. Cost Effectiveness and Resource Allocation 4: 20.

29 Over M, Revenga A, Msasaki E, Peerapatanapokin W, Gold J, et al (2007) The economics of effective AIDS treatment in Thailand. AIDS 21(Suppl 4): S105-16.

30 Walensky RP, Wood R, Ciaranello AL, Paltiel AD, Lorenzana SB, et al (2010) Scaling Up the 2010 World Health Organization HIV Treatment Guidelines in Resource-Limited Settings: A Model-Based Analysis. PLoS Med 7(12): e1000382.

31 Bendavid E, Grant P, Talbot A, Owens DK, Zolopa A (2011) Cost-effectiveness of antiretroviral regimens in the World Health Organization's treatment guidelines: a South African analysis. AIDS 25: 211–220.

32 Ciaranello AL, Lockman S, Freedberg KA, Hughes M, Chu J, et al (2011) First-line antiretroviral therapy after single-dose nevirapine exposure in South Africa: a cost-effectiveness analysis of the OCTANE trial. AIDS 25:479–492.

33 Bachmann MO (2006) Effectiveness and cost effectiveness of early and late prevention of HIV/AIDS progression with antiretrovirals or antibiotics in Southern African adults. AIDS Care 18(2): 109-120.

34 Bonnel R (2000) Costs of scaling HIV program activities to a national level in sub-Saharan Africa: Methods and estimates. Washington D.C.: World Bank.

35 Kumaranayake L, Conteh L, Kurowski C, Watts C (2001) Preliminary estimates of the cost of expanding TB, malaria and HIV/AIDS activities for sub-Saharan Africa. Geneva: Working Group 5, WHO Commission on Macroeconomics and Health.

36 Creese A, Floyd K, Alban A, Guinness L (2002) Cost-effectiveness of HIV/AIDS interventions in Africa: a systematic review of the evidence. Lancet 359: 1635-42.

37 Hogan DR, Baltussen R, Hayashi C, Lauer JA, Salomon JA (2005) Achieving the millennium development goals for health: Cost effectiveness analysis of strategies to combat HIV/AIDS in developing countries. Brit Med J, doi:10.1136/bmj.38643.368692.68

38 Floyd K, Gilks C (1997) Cost and financing aspects of providing antiretroviral therapy. In: Van Praag E, Fernyak S, Martin Katz A, eds. The implications of antiretroviral treatments. Informal Consultation. World Health Organization, Office of HIV/AIDS and Sexually Transmitted Diseases. Geneva: WHO.

39 Hogg R, Weber A, Craib K, Aslam A, O'Shaughnessy M, et al (1998) One world, one hope: the cost of providing antiretroviral therapy to all nations. AIDS 12: 2203-9.

40 Attaran A, Sachs J (2001) Defining and refining international donor support for combating the AIDS epidemic. Lancet 357: 57-61

41 Schwartländer B, Stover J, Walker N, Bollinger L, McGreevey W, et al (2001) Resource needs for HIV/AIDS. Science 292(5526): 2434-6.

42 Gutierrez J, Johns B, Adam T, Bertozzi SM, Edejer TT (2004) Achieving the WHO/UNAIDS antiretroviral treatment 3 by 5 goal: what will it cost? Lancet 364: 63-4.

43 Stover J, Korenromp E, Blakley M, Komatsu R, Viisainen K (2011) Long-Term Costs and Health Impact of Continued Global Fund Support for Antiretroviral Therapy. PLoS ONE 6(6): e21048.

44 Resch S, Korenromp E, Stover J, Blakley M, Krubiner C, et al (2011) Economic Returns to Investment in AIDS Treatment in Low and Middle Income Countries. PLoS ONE 6(10): e25310.

45 Schwartländer B, Stover J, Hallett T, Atun R, Avila C, Gouws E, et al (2011) Towards an improved investment approach for an effective response to HIV/AIDS. Lancet 377: 2031–41

46 Long EF, Brandeau ML, Owens DK (2010) The Cost-Effectiveness and Population Outcomes of Expanded HIV Screening and Antiretroviral Treatment in the United States. Ann Intern Med 153: 778-789.

47 Over M, Heywood P, Gold J, Gupta I, Hira S, Marseille E (2004) HIV/AIDS treatment and prevention in India: Modeling the costs and consequences. Washington, D.C.: The International Bank for Reconstruction and Development/ The World Bank.

48 Vijayaraghavan A, Efrusy MB, Mazonson PD, Ebrahim O, Sanne IM, et al (2007) Cost effectiveness of alternative strategies for initiating and monitoring highly active antiretroviral therapy in the developing world. J Acquir Immune Defic Syndr 46(1): 91-100.

49 Granich RM, Gilks CF, Dye C, De Cock KM, Williams BG (2009) Universal voluntary HIV testing with immediate antiretroviral therapy as a strategy for elimination of HIV transmission: a mathematical model. Lancet 373: 48–57.

50 Hontelez JAC, de Vlas SJ, Tanser F, Bakker R, Bärnighausen T, et al (2011) The Impact of the New WHO Antiretroviral Treatment Guidelines on HIV Epidemic Dynamics and Cost in South Africa. PLoS ONE 6:e21919.
