# Supplementary material for: HIV Treatment as Prevention: Modelling the Cost of Antiretroviral Treatment—State of the Art and Future Directions
Source: PLoS Med. 2012 Jul 10;9(7):e1001247. doi: 10.1371/journal.pmed.1001247 (PMC3393674; doi:10.1371/journal.pmed.1001247)
Supplement: Text S2 — The algebra of flexible cost functions. (PDF) [file pmed.1001247.s002.pdf]

Text S2: The algebra of flexible cost functions

Supporting information for

HIV Treatment as Prevention: Modelling the Cost of Antiretroviral Treatment- State of the Art and Future Directions

Gesine Meyer-Rath[1,2] and Mead Over[3]

[1] Center for Global Health and Development, Boston University, Boston, US
[2] Health Economics and Epidemiology Research Office (HE^{2}RO), Department of Medicine, Faculty of Health Sciences, University of the Witwatersrand, Johannesburg, South Africa
[3] Center for Global Development, Washington DC, US

A “cost function” is a mathematical function that defines the relationship between an output produced, here defined as patient-years (or -months) of antiretroviral therapy (ART), and the cost of the resources consumed in that production. A cost function, either explicit or implicit, is a necessary component of any estimate of the costs and benefits of ART service delivery. This appendix gives supporting details on the mathematical distinction between two classes of cost functions, which we refer to as “accounting identity” cost functions and “flexible” cost functions. Our purpose is not to elevate one of these types of cost functions over the other in all situations, but to distinguish them as distinct approaches to projecting costs, each of which has its strengths and weaknesses. In modelling practice, the most useful cost function might be one or the other or, in some cases, a hybrid of the two.

For those unversed in economics, the main text of this paper attempts to introduce readers to cost function ideas that originate in that discipline, while avoiding economics jargon. However, those readers who build or use mathematical or cost models will want a more substantial introduction to the literature which undergirds our discussion than we have room for in the main text. We use the additional space available to us in this appendix to expand somewhat our discussion of the two types of cost functions and to provide references to some of the seminal work from which the intellectual concepts of cost functions have grown. To this end, this appendix uses more economic terminology and mathematical notation than the main text.

We define an “accounting identity cost function” as one that is constructed by enumerating all of the inputs to a production process, multiplying the number of units required of each input by that input’s unit cost, and summing across all of these products to arrive at a total cost. The accounting identity approach relies on a detailed understanding (or assumptions) about every step of the production process. Assembling the cost from all the inputs consumed in a time period such as a year yields total cost which can be related to the total number of units of output produced during that year. Assembling the cost per unit of output of all the inputs yields average total cost. Relating either total cost constructed in this way or average total cost to output gives an accounting identity cost function. The salient features of the accounting identity approach to constructing a cost function are summarised in the first column of Table S2. Depending on the amount of detail it captures, an accounting identity cost function can be simple or complex.

In contrast, a “flexible cost function” ignores the details of the production process, instead treating it as a “black box”. This function characterises the relationship between total or average cost and output by a functional relationship which is influenced by a set of determinants such as input prices, environmental and policy variables and managerial incentives. The second column of Table S2 contrasts the attributes

of a flexible cost function to those of an accounting identity cost function. Depending on the functional form chosen, a flexible cost function can also be simple or complex.

Both of these ideal type cost functions have antecedents in the economics literature. The accounting identity cost function is philosophically descended from the “engineering production functions” introduced to the economics literature in 1949, the point of which was to model with as much detail as possible the actual processes by which a firm converts inputs to output[1]. Accounting identity cost functions typically assume a linear production structure such as that used in input-output analysis as originally developed by Leontief[2]. A prominent microeconomics textbook presents an accounting identity cost function in order to contrast it to a flexible cost function[3].
The concept of the flexible cost function derives from the need in economics to characterise the complexity of real-world production relationships with a relatively parsimonious mathematical relationship between inputs and outputs. Douglas first developed and applied to empirical data such a summative description of production in what is now called the Cobb-Douglas production function[4]. Although parsimonious and more flexible than an accounting identity, the Cobb-Douglas production function and its associated cost function have been found to be insufficiently flexible to describe some production processes. This realisation led to the development and application to health care production of a portfolio of more flexible functions, including a generalisation of the Cobb-Douglas function which is called the translog cost function[5,6]. A flexible cost function can be simple, but increased flexibility usually entails more nonlinear terms in the function and thus is associated with greater complexity.

In the literature on the cost of disease, Scitovsky and Over have drawn the distinction between “normative” and “positive” approaches to cost estimation[7]. They define a normative approach as one that aims to estimate how much it *should* cost to treat a given disease (according to a “norm”, however defined), while a positive approach is one which aims to estimate how much a country or health system is *actually* spending on the disease. Our distinction between the accounting identity cost function and the flexible cost function is related to, but not identical to, the normative/positive distinction. Typically those taking a normative approach use an accounting identity cost function, because it allows them to specify all the details of the recommended medical treatment protocol, the costs of which they wish to capture. On the other hand, some applications of the accounting identity approach might adopt a positive perspective by collecting and assembling information on all the detailed cost elements of an actual observed production process into an accounting identity cost function. The flexible cost function is closely associated with the empirically based or “positive” approach to costing. Assembling data on the total cost and total output of a sample of ART facilities and estimating a flexible cost function by multiple regression is the quintessential positive approach to cost function estimation.

The health economics literature is replete with empirical (i.e. positive) estimates of flexible cost functions. Monographs by Feldstein and Barnum and Kutzin estimate and draw policy inferences from hospital cost functions for, respectively, developed and developing countries[8,9]. Over calibrates a flexible cost function to basic data on the cost of primary health care delivery in Africa[10], while Guiness, Kumaranayake and Hanson fit such a function to HIV prevention services in India[11]. To our knowledge no flexible cost function has yet been fit to ART delivery in either a developed or developing country. While parametric flexible cost functions are likely to be most useful to modellers, the non-parametric approach to analysing the relationship of cost to output is even more flexible, as recently shown in a study of the US hospital industry[12].

Box 1 and Tables S2 and S3 provide a detailed comparison of the characteristics of an accounting identity cost function to those of a flexible cost function. Box 1 shows graphically how the flexible cost function is able to represent non-linear relationships between input prices (Case A) or output quantity

(Case B) and total cost in contrast to the more rigidly linear relationship typically inherent in an accounting identity function. (Note that the linear relationship between prices and cost usually embodied in an accounting identity cost function also typifies a cost function that is dual to a Leontief production function which rigidly prescribes the ratios in which inputs must be used[13]). The figures show how the projections of a cost accounting identity and a cost function will typically diverge as an input price (Figure S1) or the scale of production (Figure S2) diverges from its current value. Note that the crucial difference is in the second partial derivative in each case. In both Case A and B, the accounting identity cost function assumes the relevant second derivative to be zero, while the flexible cost function allows it to adopt the value that best fits observed or hypothetical curvature in the relevant relationship.

Box 1. Relationship between the cost accounting identity and the flexible cost function

A. The cost accounting identity tends to over-estimate costs at prices different from the current observed prices, because economic agents will respond to price changes by economising on higher priced inputs.

- Define $TC,\ Q,\ X_i, p_i,\ Z_m$ as national levels of total cost, output, quantity of input used per patient, price of input *i*, policy determinant *m* observed in a given year. (The year subscript is suppressed.)
- Cost accounting identity

$$TC_{AI} = Fixed\ Cost +\ AVC \cdot Q \text{ where } AVC = \sum_{i=1}^{N}(p_i \cdot X_i)$$

$$\frac{\partial TC}{\partial p_i} = Q \cdot X_i > 0\ ,\ \frac{\partial^2 TC}{\partial\, p_i^2} = 0$$

- Cost function

$$TC_F = f(p_i,\ Z_m,\ Q) \text{ where } \frac{\partial TC}{\partial p_i} > 0\ ,\ \frac{\partial^2 TC}{\partial\, p_i^2} < 0$$

- In Figure S1 the cost accounting identity and the flexible cost function give the same estimate of total cost at the current price of input *i*, p_i*, but their projections diverge at the larger price of p_i', where the accounting identity's linear form predicts total cost TC_{AI}, while the flexible cost function predicts the smaller total cost, TC_F.

Figure S1: Impact of input prices on total cost. While the accounting identity cost function is linear in the price of each input, the flexible cost function captures economising behaviour and projects lower total cost at prices different than those currently observed.

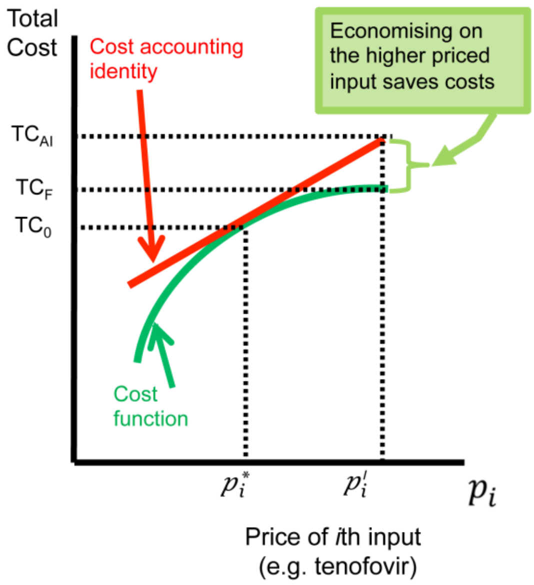

Box 1 (continued)

B. The cost accounting identity will over- or under-estimate costs at levels output different from the current observed level of output. For example, if the current level of output is adjusted to local demand and production capacity, linear extrapolation to different output levels will underestimate total cost.

- Define TC, Q, p_i, Z_m as above
- Cost accounting identity

$$TC_{AI} = Fixed\,Cost + AVC \cdot Q \text{ where } AVC = \sum_{i=1}^{N}(p_i \cdot X_i)$$

$$\frac{\partial TC}{\partial Q} = AVC > 0\,,\ \frac{\partial^2 TC}{\partial\, Q^2} = 0$$

- Cost function

$$TC_F = f(p_i, Z_m, Q) \text{ where } \frac{\partial TC}{\partial Q} > 0\,, \frac{\partial^2 TC}{\partial\, Q^2} > 0 \text{ or } \frac{\partial^2 TC}{\partial\, Q^2} < 0$$

- In Figure S2 the cost accounting identity and the flexible cost function give the same estimate of total cost at the current output level Q*, but their projections diverge at the larger output Q', where the accounting identity's linear form predicts total cost TC_{AI}, while the flexible cost function predicts the larger total cost, TC_F.

Figure S2: Impact of scale on total cost. While the accounting identity cost function is linear in the quantity of output, price of each input, the flexible cost function can incorporate economies or diseconomies of scale

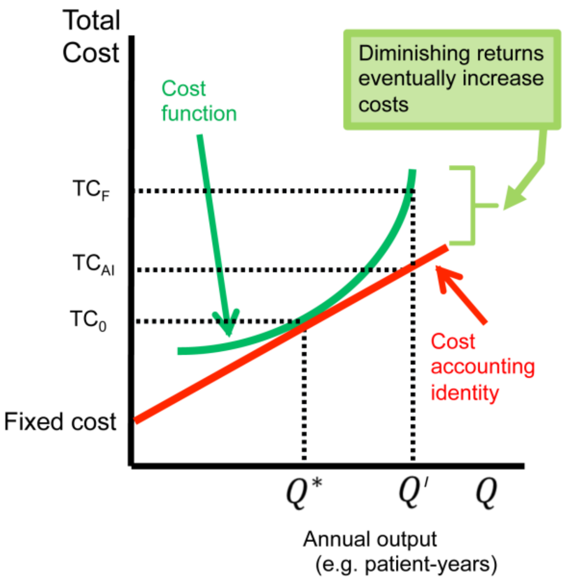

Table S2 presents a comparison of the accounting identity cost function to the flexible cost function for an individual facility. The table details how the accounting identity is constructed by assembling information on the input quantities required to treat a single patient, potentially distinguishing these estimates by types of patient. In principle, total costs at a facility are the sum of total fixed costs (which are independent of the number of patients treated) and total variable costs (which increase monotonically with the number of patients treated). Accounting identity cost functions, to be complete, should include both fixed and variable costs, but analysts often either omit fixed cost at the facility level or fold the fixed cost into the variable cost, assumptions which we think are difficult to justify in the case of antiretroviral treatment.

Table S2: Cost functions for an individual facility for one time unit (month or year)

	**Accounting identity cost function**	**Flexible cost function**
Output of the kth facility	$\boldsymbol{q_k}$ patient-months (or years) of ART is the sum over J types of patients<br>$q_k = \sum_{j=1}^{J} q_{jk}$	
Ratios of inputs	Input ratios are typically assumed to be determined by the technology and thus fixed or fixed with a patient category	Input ratios can vary in response to price changes
Efficiency of production	Typically effort and quality are assumed to conform to established norms	Can vary in response to incentives, both financial and non-financial
Fixed cost of the kth facility	Typically neglected or modelled as the one-time cost of facility construction	Typically incurred per time unit, either varies by facility, fc_k, or the same across all facilities, *fc*
Variable cost of the k'[h] facility	Might vary by drug regimen, health state, time on treatment or other fixed attribute of the patient or facility	Might vary by numbers of patients, provider incentives, experience, scope of production or other managerial or programme attributes
Total cost for a single facility, facility k, with J patient types	$\begin{matrix} Total \\ cost_k \end{matrix}(tc_k) = \begin{matrix} Total \\ fixed(tfc) \\ cost \end{matrix} + \begin{matrix} Total \\ variable(tvc_k) \\ cost_k \end{matrix}$<br><br>where *tfc* = *0* and tvc_k is the sum over *J* patient types:<br>$tvc_k = \sum_{j=1}^{J} avc_{jk}\, q_{jk}$<br>and avc_{jk} is the sum over the I_j inputs for patient type, *j*:<br>$avc_{jk} = \sum_{i \in I_j} p_i x_i$<br>where p_i and x_i are the price and quantity of the *i'th* input and inputs include drugs, reagents, personnel, etc.. If avc_{jk} is constant and equal to avc_j over all facilities, tc_k simplifies to:<br>$tc_k = \sum_{j=1}^{J} avc_j\, q_{jk}$	$\begin{matrix} Total \\ cost_k \end{matrix}(tc_k) = \boldsymbol{f}(p_i, z_{mk}, q_{jk})$<br><br>where *f(.)* is a flexible function of the prices of all inputs, the quantities of all patient categories and a set of environmental, contextual and policy variables, z_{mk}, for *m = 1,…, M*.<br><br>The parametric function f(.) is selected to capture plausible characteristics of the problem at hand and then fitted to available data. Functional forms used in the economics literature include polynomial functions in q_k, or in its logarithm and second-order Taylor series approximations of arbitrary functions of output and of the input prices.
Average cost (or "unit cost") per patient-month (or patient-year) of ART for a single facility, facility k	$atc_k = \frac{tc_k}{q_k} = avc_k = \sum_{j=1}^{J} avc_{jk} \frac{q_{jk}}{q_k}$<br><br>The fraction ${q_{jk}}/{q_k}$ is the share of the jth patient type in the total patient load for facility *k* in that year. Thus avc_k, is a weighted average of the average variable costs of the *J* patient types. Typically avc_k varies with change across facilities or time in the input prices or in the mix of patient types or inputs for a specific patient type.	$atc_k = \frac{tc_k}{q_k} = \frac{f(p_i, z_{mk}, q_{jk})}{q_k}$<br><br>Functions which allow economies or diseconomies of scale include:<br>a) $atc_k = \frac{A}{q_k} + B(p_i, z_{mk}, q_{jk}) - Cq_k + Dq_k^2$<br>b) $atc_k = A(p_i, z_{mk}, q_{jk})\, {q_k^{\sigma}}/{q_k}$<br>c) $\ln(atc_k) = A + B\sum ln(p_i) + C\sum\sum ln(p_i)ln(p_j) + (\sigma - 1)\, ln(q_k)$<br>where *σ* is the scale elasticity and the "total fixed cost" is defined as the value of atc_k when $q_k = 1$.

Table S3 presents a comparison of the accounting identity cost function to the flexible cost function for an aggregate across many facilities, such as might be appropriate for estimating regional, national or global costs of ART. In principle total regional cost is defined as the sum of the costs across all facilities, including the costs of all patients treated at each facility. Both the aggregate accounting identity cost function and the aggregate flexible cost function can be formed by adding together the facility specific cost functions across all individual facilities. If facility-specific estimates were available by either method, the analyst could construct the aggregate simply by assembling them. However, as we detail in the paper, data on cost and output of a large enough sample of ART facilities are not yet available even in South Africa, where treatment enrolment has reached 1.7 million patients and is now delivered in more than 2000 distinct facilities.

Table S3: Cost functions for a country for one time unit (month or year)

	Accounting identity cost function	Flexible cost function
Output of the country for one unit of time	$\boldsymbol{Q}$ patient-months (or years) of ART is the sum over K facilities or over all J patient types<br>$Q = \sum_{k=1}^{K} q_k = \sum_{k=1}^{K}\sum_{j=1}^{J} q_{jk} = \sum_{j=1}^{J} q_j$<br>where q_j is defined as the national total across all facilities of patients in category *j*	
Fixed cost for the nation	Typically neglected or modelled as the one-time cost of facility construction	Typically follows from the functional form for facility specific costs, but could include national level fixed cost
Variable cost per patient	avc_j, if patient mix and prices are assumed constant across facilities	Not independently defined
Total cost for country with J patient types	Total cost for the country, *TC*, is the sum of all the individual facility costs, tc_k<br>$TC = \sum_{k=1}^{K} tc_k = \sum_{k=1}^{K}\sum_{j=1}^{J} avc_{jk}\, q_{jk}$<br>But if the average variable cost of patient type *j* is assumed to be the same at all facilities, $avc_{jk} = avc_j$, then total cost no longer depends on facility level information and can be written as:<br>$TC = \sum_{j=1}^{J} avc_j \sum_{k=1}^{K} q_{jk} = \sum_{j=1}^{J} avc_j\, q_j$	Total cost for the country, *TC*, is the sum of all the individual facility costs, tc_k<br>$TC = \sum_{k=1}^{K} tc_k = \sum_{k=1}^{K} \boldsymbol{f}(p_i, z_{mk}, q_{jk})$<br>where *f(.)* is a flexible function of the prices of all inputs, the quantities of all patient categories and a set of environmental, contextual and policy variables, z_{mk}, for *m = 1,…, M*.
Average cost (or "unit cost") per patient-month (or patient-year) of ART for entire country	$ATC = {}^{TC}/_{Q} = \sum_{j=1}^{J} avc_j \frac{q_j}{Q}$<br><br>The fraction ${}^{q_j}/_{Q}$ is the share of the th patient type in the total patient load for the country in that time period. Thus, national average cost per time period, *ATC*, is a weighted average of the average costs of the J patient types. Characteristics of the patient delivery process, such as scale, scope, experience and incentives have no effect on average cost.	$ATC = \sum_{k=1}^{K} \frac{\boldsymbol{f}(p_i, z_{mk}, q_{jk})}{Q}$<br><br>with estimates of its parameters at the facility level, any of the above functional forms for a facility-level average cost function can be aggregated to the national level. One of the simplest to use with little data is function b), which aggregates to:<br>$ATC = \sum_{k=1}^{K} \bar{A}\frac{q_k^{\sigma}}{Q} = \bar{A}\sum_{k=1}^{K}\frac{q_k}{Q}\frac{q_k^{\sigma}}{q_k}$<br>where σ is the elasticity of scale and $A(p_i, z_{mk}, q_{jk})$ is assumed constant at $\bar{A}$.

Table S3 also shows how an aggregate cost function can be approximated when neither type of cost function is yet available at the facility level. Following the accounting identity approach, the analyst can assume that the average variable cost (avc) of treating one person is constant within patient category across all sizes and types of facility (i.e. assume that $avc_{jk} = avc_j$ for all *j=1,…,J* patient types and all *k=1,…,K* facilities*)*. Following the flexible cost function approach when empirical data on the curvature of the flexible cost function is not yet available requires the analyst to adopt a plausible parsimonious parameterisation of the flexible function, much as the epidemiologist might adopt such a parameterisation

of a sexual mixing matrix or an infectivity profile, and then perform sensitivity analysis with respect to these selected parameters.

The example presented in the table and in the worked application to the Granich et al[14] projections presented in the text supposes that the individual facility has a total cost function with returns to scale σ of the form[a]:

$$tc_k = A(p_i, z_{mk}, q_{jk})q_k^{\sigma}$$

This specification asserts that a ten per cent increase in output is associated with a 10σ per cent increase in cost. The parameter σ is referred to as the elasticity of total cost with respect to output. With a value of σ less than one, any given percentage increase in output is associated with a less than proportional increase in total cost, so the production technology benefits from increasing returns to scale. Production technologies characterized by values of σ equal to or greater than one are described as having constant or decreasing returns to scale, respectively.

Adopting the (over)simplifying assumption that the combined effect of local input prices, p_i, environmental, contextual and policy determinants of efficiency, z_{mk}, and mix of patient categories, q_{jk}, are constant across all facilities, the function of these variables which multiplies facility output collapses to a constant, $\bar{A}$, as follows:

$$A(p_i, z_{mk}, q_{jk}) = \bar{A}$$

With this assumption, we can divide through the facility-specific total cost function by the facility's total patient load, q_k, to obtain the facility's average cost function:

$$atc_k = \bar{A}\, {q_k^{\sigma}}/{q_k} = \bar{A} q_k^{\sigma-1}$$

A value of σ smaller than one thus corresponds to an average total cost function that declines with increasing output. A value of σ equal to one corresponds to the special case of constant returns to scale in which the average total cost of a facility is constant and equal to $\bar{A}$.

Adding these facility-specific flexible total cost functions across all K facilities gives an aggregate total cost function,

$$TC = \sum_{k=1}^{K} tc_k = \sum_{k=1}^{K} \bar{A} \cdot q_k^{\sigma} \qquad \text{(S1)}$$

Dividing aggregated total cost, TC, by total patients in the entire country, Q, defined as $Q = \sum_{k=1}^{K} q_k$, gives an expression for the average total cost of AIDS treatment in that country (and that time period):

$$ATC = \sum_{k=1}^{K} \bar{A} \frac{q_k^{\sigma}}{Q}$$

[a] In the following expression and in the tables and figures, all quantities are measured within a specified time period such as a month or, more typically, a year and refer to the amounts of inputs, or outputs that are processed during that time period and to the average values of the prices and other determinants that obtain during that time period. This accords with an approach that Scitovsky and Over dub the "prevalence-based" approach to estimating the cost of a disease which assigns to a given time period only the costs expended during that time period[7]. This approach contrasts with the "incidence-based" approach to costing a disease, which attributes to any year the future discounted cost of all cases that begin that year.

Since the sum of facility specific output, q_k, over all facilities equals total national output Q, it is useful to rewrite this function as:

$$ATC = \sum_{k=1}^{K} \frac{q_k}{Q} \bar{A} \frac{q_k^{\sigma}}{q_k} = \sum_{k=1}^{K} \frac{q_k}{Q} atc_k$$

where the fraction $q_k/_Q$ represents the share of facility k's output in the national total. This expression shows that average total cost can be expressed as the weighted average of the facility specific average costs, where the weights are the K output shares, $q_k/_Q$. Except in the special case where σ equals one, cost is non-linearly related to output at the facility level, so the behaviour of national total cost cannot be modelled accurately without information about how total output is distributed among the K facilities.

For our worked example that shows the effect of potential scale economies on the cost of a large scale-up of ART, we calibrate equation S1 to South African data on the 1,095 health care facilities that were accredited for ART and the number of patients enrolled at those facilities in mid-2010. Sorting facilities by the size of their patient load from largest to smallest, the facility index *k* can be interpreted as the rank of a facility in the size distribution of all facilities. Unlike many mature size distributions, the early-2010 sizes of South African ART facilities ranked by number of ART patients is not uniformly log-linear, but it can be closely approximated by a piece-wise linear spline with knots at 50 and 400. The following linear spline in the logarithm of facility rank explains 94 per cent of the variation in the logarithm of the facility's number of enrolled ART patients.

$$\widehat{\ln(q_k)} = 9.8 + \begin{cases} -0.323 \cdot \ln(k) & if\ k \leq 50 \\ 1.59 - 0.729 \cdot \ln(k) & if\ k > 50\ and\ k \leq 400 \\ 3.63 - 1.070 \cdot \ln(k) & if\ k > 400 \end{cases} \quad \text{(S2)}$$

Expressed in the original units, the piecewise approximation to the empirical size-rank distribution can be written as:

$$\hat{q}_k = e^{9.8} \cdot \begin{cases} k^{-0.323} & if\ k \leq 50 \\ k^{-0.323} \cdot \left(\frac{k}{50}\right)^{-0.406} & if\ k > 50\ and\ k \leq 400 \\ k^{-0.323} \cdot \left(\frac{k}{50}\right)^{-0.406} \cdot \left(\frac{k}{400}\right)^{\rho} & if\ k > 400 \end{cases} \quad \text{(S3)}$$

where ρ can be varied in order to simulate scaling up treatment access, while holding constant the capacity of the largest 400 ART treatment facilities. Increasing ρ from its fitted value of -5.4 to the value -0.34 simulates increasing the sizes of the smallest 695 facilities to accommodate the 1.5 million patients enrolled by mid-2010. According to national estimates, the total cost of ART in mid-2010 was approximately US$1.387 billion[15]. Substituting this amount for TC on the left of equation S1 and substituting S3 for q_k with ρ set to -0.34 gives an equation in the two unknowns $\bar{A}$ and σ:

$$1.387 \cdot 10^9 = \bar{A} \cdot \sum_{k=1}^{K} \hat{q}_k^{\sigma} \quad \text{(S4)}$$

Solving this equation for $\bar{A}$ for various values of σ between 0.5 and 1.0 yields a family of average cost functions for the individual South African ART treatment facility with the parameter values given in Table S4 and presented in Figure S3.

Table S4. Calibration of flexible cost function to South African data for 2010/11

	Value of σ	Value of (σ – 1)	Value of $\bar{A}$	
	Percent increase in total cost associated with a 1% increase in output (Scale elasticity)	Percent decrease in average total cost associated with a 1% increase in output	Cost of using an entire ART facility to treat a single patient	
			Derived from Meyer-Rath et al	Deflated to match Granich et al costs
Constant returns to scale	1.0	0	$924	$800
Increasing returns to scale	0.9	-0.1	$1,976	$1,711
	0.8	-0.2	$4,187	$3,625
	0.7	-0.3	$8,791	$7,611
	0.6	-0.4	$18,296	$15,840
	0.5	-0.5	$37,763	$32,695

Source: Last column are authors' calculations using equation S2-3.

Figure S3. Family of average cost curves derived from South African cost data (Source: authors' calculations using equation S2-3).

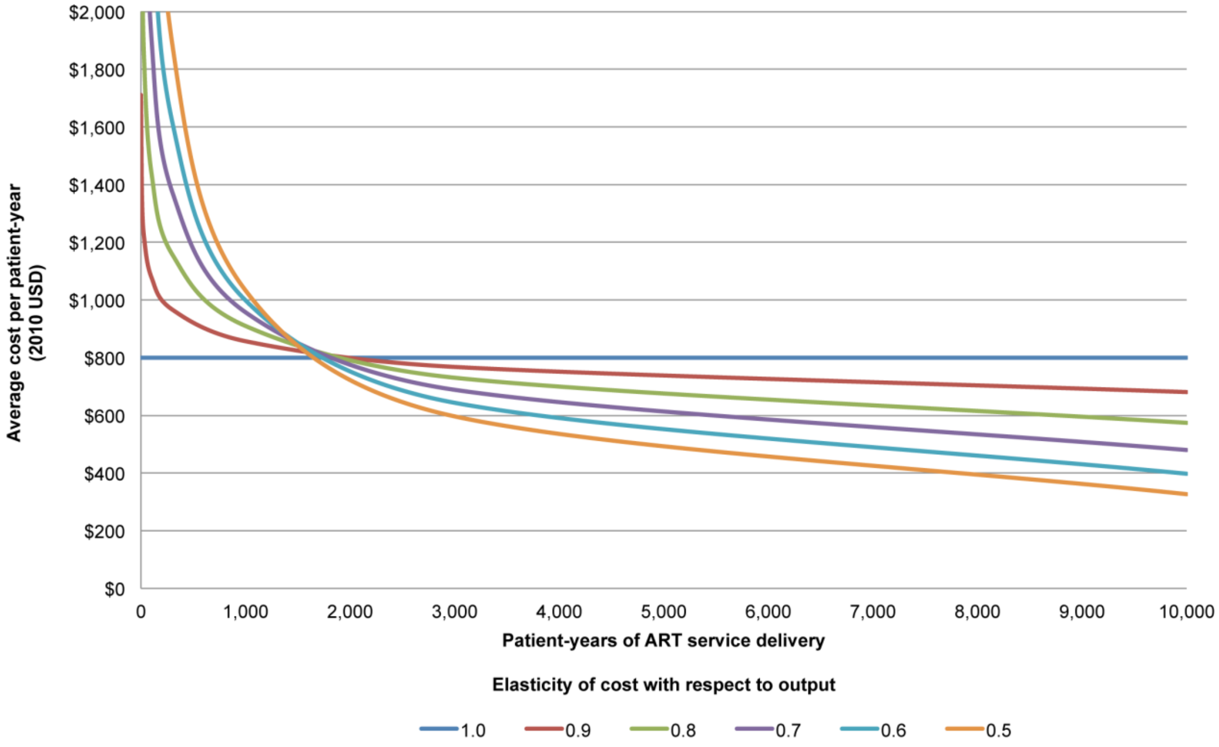

To examine the sensitivity of the Granich et al projections to a range of alternative elasticities of scale, we first need to produce a projection under constant returns to scale that closely matches the central projection published in that paper[14]. Panel E of Figure 4 and Figure 6 in Granich et al shows two scenarios, the universal test and treat (UTT) scenario and a scenario representing the full implementation of the latest WHO ART guidelines suggesting treatment initiation at a 350 CD4 count in South Africa,

which we refer to as the WHO scenario. By close examination of Figure 6 in Granich et al we estimate the peak expenditures for the two scenarios in 2016 of the projections as $3.28 and $1.4 billion US 2010 dollars respectively. Panel E of Figure 4 in Granich et al gives the numbers of adults on treatment denominated in proportions of the adult population. Since the South African adult population is estimated at 35 million for 2016, we can apply the proportions read from the figure to estimate that the paper is projecting about 4.1 million people on treatment under the UTT scenario and about 1.75 million people on treatment under the WHO scenario. These estimates are consistent with the assumption that the authors are holding the average cost per patient-year constant at about $800, which in turn is consistent with their stated assumptions that first-line and second-line ART cost respectively $727 and $3290 per patient-year and a constant 3 per cent of patients move to second-line each year. The starting point for our simulations of the effect of scale on the cost of the UTT strategy is to simply divide Granich et al's cost projections by $800 in order to arrive at a time path of the number of patients to be treated under that strategy. All of our simulations of the future cost of the UTT strategy use this single projection of the total number of patient-years of treatment to be offered in future years.

Next we posit a change in the size distribution over time. We do this first for the period from 2010 until the year 2016, in which Granich et al predict the total number of patients and total ART expenditure will reach a maximum[14]. For this expansion phase, we posit a linear increase in the number of facilities delivering ART from their 2010 level of about 1,095 to enough facilities to reach all the patients envisaged by the UTT policy. We assume that 6,000 facilities will be needed at peak intensity. For the second phase, we suppose that most facilities that have been accredited for ART delivery will remain active, but reduce the number of patients being served. So we model the period after 2016 as one of declining numbers of facilities, on an ogival trajectory to return to only 1,000 facilities delivering ART by the year 2100. Figure 3 of the main text and Figure S4 below illustrate various aspects of the projected size distribution of facilities through the year 2050.

Figure S4. Number of facilities delivering ART and the number of patients served by the smallest ART facility: 2010-2050

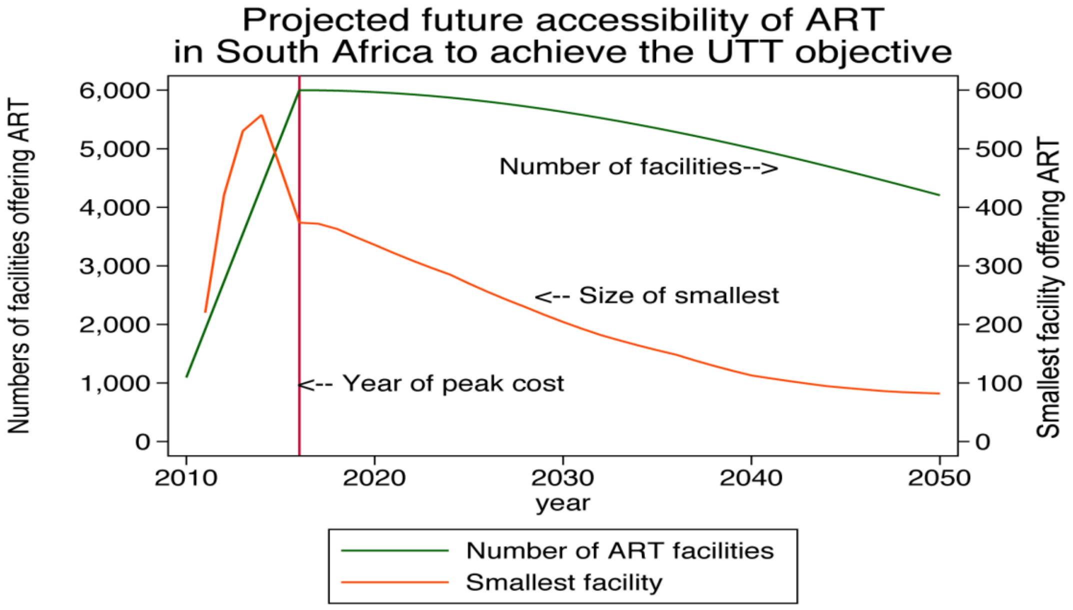

During the contraction phase of the treatment programme, we assume that the size distribution of facilities will have matured and more closely follow Zipf's law as given by the equation:

$$\hat{q}_{kt} = \pi_t \cdot e^{9.8} \cdot k^{\rho} \qquad \text{(S5)}$$

where the parameter π_t allows all facilities to decrease their patient loads once the total number of patients has declined to under one million. The parameter π_t is thus defined as follows:

$$\pi_t = \min \left[1.0, \frac{Number\ \ of\ enrolled\ \ ART\ patients\ \ in\ year\ \ t}{1{,}000{,}000}\right] \qquad \text{(S6)}$$

For any year after 2016 we now have the number of facilities, the number of patients and the deflation proportion π_t. From these quantities we can solve for value of the parameter ρ that is specific to each year and thus compute the entire rank-size distribution for each year.

In order to use the family of average cost curves derived from actual 2010/11 South African costs, where average cost per patient was $924, we must first deflate those cost curves down to the $800 average cost used by Granich et al. The last column of Table S5 presents those deflated values of the A parameter.

We now have all the pieces necessary to project the total cost of the UTT strategy under a variety of assumptions regarding the elasticity of scale. Table S5 and Figure S5 present the results of these calculations. When the scale elasticity is set to 1.0, the projection model embodies the constant returns to scale assumption and mimics the original projection for the UTT option in Granich et al[14]. As the scale elasticity parameter is decreased, corresponding to a steeper average cost curve and therefore higher costs for smaller facilities, both the peak programme cost and the cumulated cost of the UTT programme also increase. If the true elasticity of scale is 0.7 rather than 1.0, the peak cost of UTT will be 26% larger than in Granich et al.'s projection, while the total undiscounted cumulated cost through 2016 will be 40% larger than projected. Furthermore, if economies of scale are even greater, with an elasticity of scale of 0.5, the total cost in 2016 would rise to US$5.1 billion, 45% larger than the base case, and the total accumulated undiscounted cost through the year 2050 would rise by 75% to US$131 billion.

Table S5. Impact on peak-year and cumulated cost of a Universal Test and Treat policy in South Africa of alternative assumptions regarding economies of scale in ART service delivery

	Value of σ	Costs of Universal Test and Treat policy		
	Per cent increase in total cost associated with a one per cent increase in output (Scale elasticity)	Peak cost in billions of USD	Total cumulated cost without discounting in constant 2010 USD: Total cost in billions of USD	Total cumulated cost without discounting in constant 2010 USD: Per cent of total above constant returns to scale
Constant returns to scale	1.0	$3.5	$74.6	0.0%
Increasing returns to scale	0.9	$3.8	$83.6	12.0%
	0.8	$4.1	$93.6	25.4%
	0.7	$4.4	$104.8	40.4%
	0.6	$4.7	$117.2	57.0%
	0.5	$5.1	$131.0	75.4%

Source: First row based on Granich et al[14]. Other rows are authors' calculations as described in the paper and in this appendix.

Figure S5. Impact of scale economies on the future cost of the UTT option

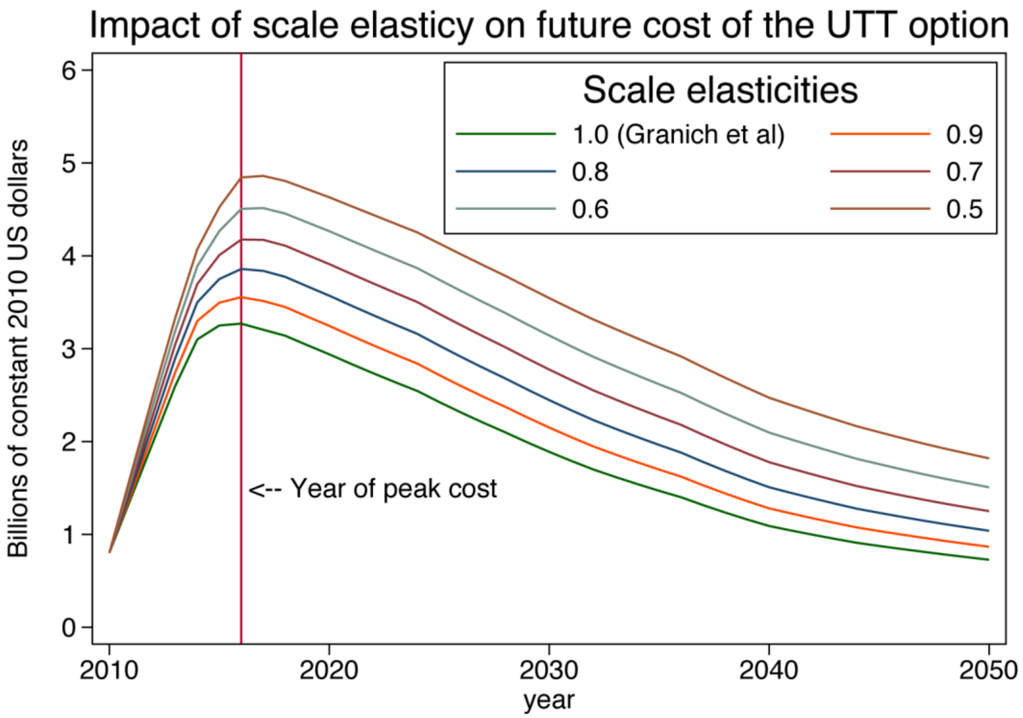

Granich et al present not only their central projections, which we have discussed here, but also upper and lower bounds around that central scenario[14]. Since the assumptions they vary to produce these upper and lower bounds include the price of ART dugs and unspecified epidemiological parameters, scale considerations will shift all three projections upward, raising the possibility that the worst case projections could be much higher than we have estimated here.

Our result that a flexible cost function would predict higher future ART costs in South Africa than would an accounting identity cost function depends on several assumptions. Most important of these is our assumption of the future change in the size distribution of facilities that would be required to accommodate all 4.1 million patients in the peak year of expansion, an assumption we describe in Figures 3 and S4 and in the accompanying exposition. Since our purpose here is only to give an example of the application of a flexible cost function, we stop short of performing sensitivity analysis of the results to alternative future size distributions. However if it is possible to imagine treating all 4.1 million patients in a small number of gigantic facilities similar to the largest currently active in South Africa, the same elasticity of scale assumption would project smaller costs than in the Granich et al paper. On the other hand, if the South African HIV programme finds that in order to maintain patient adherence at the largest scale it must distribute ART service delivery to even smaller and more numerous facilities than we have posited, the same elasticity assumption would produce even larger future costs. Our point is that to be plausible and useful to policy makers, models of the costs and benefits of ART over the long run must grapple with the question of how those services will be delivered and how changes over time in the determinants of cost and quality will affect that delivery. Adopting well-specified flexible cost functions is likely to be an integral part of that effort.

References

1. Chenery H (1949) Engineering production functions The Quarterly Journal of Economics 63(4): 507-531.

2. Leontief W (1941) The structure of the American economy. Cambridge: Harvard University Press

3. Varian H (1984) Microeconomic Analysis. Norton. Figure 1.24, p.45.

4. Cobb CW, Douglas PH (1928) A theory of production. American Economic Review (Supplement) 139-172.

5. Varian, op. cit., p. 181.

6. Bilodeau D, Crémieux PY, Ouellette P (1984) Hospital cost function in a non-market health care system. Review of Economics and Statistics 82(3): 489-498.

7. Scitovsky A, Over M (1988) AIDS: costs of care in the developed and the developing world. AIDS 2(Suppl 1): S71-S81.

8. Feldstein M (1981) Hospital Costs and Health Insurance. Cambridge, Ma.: Harvard University Press.

9. Barnum H, Kutzin J (1993) Public Hospitals in Developing Countries. Baltimore: Johns Hopkins University Press.

10. Over M (1986) The effect of scale on cost projections for a primary health care program in a developing country. Social Science and Medicine 22(3): 351-360.

11. Guiness L, Kumaranayake L, Hanson K (2007) A cost function for HIV prevention services: is there a ‘u’ shape? Cost Effectiveness and Resource Allocation 5(13)

12. Wilson P, Carey K (2004) Nonparametric analysis of returns to scale in the US hospital industry. Journal of Applied Economics 19: 505-524.

13. Diewert WE (1971) An application of the Shephard duality theorem: A generalized Leontief production function. Journal of Political Economy 79(3).

14. Granich RM, Gilks CF, Dye C, De Cock KM, Williams BG (2009) Universal voluntary HIV testing with immediate antiretroviral therapy as a strategy for elimination of HIV transmission: a mathematical model. Lancet 373: 48–57.

15. Meyer-Rath G, Brennan A, Long L, Fox M, Rosen S (2011) National ART Cost Model, South Africa. Health Economics and Epidemiology Research Office, Boston University/ University of the Witwatersrand, Johannesburg.
